# A novel small molecule Enpp1 inhibitor improves tumor control following radiation therapy by targeting stromal Enpp1 expression

**DOI:** 10.1038/s41598-024-80677-8

**Published:** 2024-12-02

**Authors:** Jason R Baird, Alejandro F Alice, Roland Saito, Qingqing Chai, Minhua Han, Cindy Ng, Stephanie Han, Beth Fernandez, Sarah Ledoux, Johannes Grosse, Alan J Korman, Megan Potuznik, Venkatesh Rajamanickam, Brady Bernard, Marka R Crittenden, Michael J Gough

**Affiliations:** 1 Earle A. Chiles Research Institute, Robert W. Franz Cancer Center, Providence Portland Medical Center, 4805 NE Glisan St, Portland, OR 97213 USA; 2 VIR Biotechnology Inc, 1800 Owens Street, Suite 900, San Francisco, CA 94158 USA; 3 The Oregon Clinic, Portland, OR 97213 USA

**Keywords:** Cancer microenvironment, Tumour immunology, Cancer therapeutic resistance, Radiotherapy

## Abstract

The uniqueness in each person’s cancer cells and variation in immune infiltrates means that each tumor represents a unique problem, but therapeutic targets can be found among their shared features. Radiation therapy alters the interaction between the cancer cells and the stroma through release of innate adjuvants. The extranuclear DNA that can result from radiation damage of cells can result in production of the second messenger cyclic guanosine monophosphate–adenosine monophosphate (cGAMP) by cyclic GMP-AMP synthase (cGAS). In turn, cGAMP can activate the innate sensor stimulator of interferon genes (STING), resulting in innate immune activation. Ectonucleotide pyrophosphatase/phosphodiesterase 1 (Enpp1) is a phosphodiesterase that can be expressed by cancer cells that can degrade cGAMP, thus can decrease or block STING activation following radiation therapy, impairing the innate immunity that is critical to support adaptive immune control of tumors. We observed that many human and murine cancer cells lack Enpp1 expression, but that Enpp1 is expressed in cells of the tumor stroma where it limits tumor control by radiation therapy. We demonstrate in preclinical models the efficacy of a novel Enpp1 inhibitor and show that this inhibitor improves tumor control by radiation even where the cancer cells lack Enpp1. This mechanism requires STING and type I interferon (IFN) receptor expression by non-cancer cells and is dependent on CD8 T cells as a final effector mechanism of tumor control. This suggests that Enpp1 inhibition may be an effective partner for radiation therapy regardless of whether cancer cells express Enpp1. This broadens the potential patient base for whom Enpp1 inhibitors can be applied to improve innate immune responses following radiation therapy.

**Supplementary Information:**

The online version contains supplementary material available at 10.1038/s41598-024-80677-8.

## Introduction

The characteristics of cancer varies significantly according to the originating cell and tissue, and varies between patients within a histological origin. Yet, conventional therapeutic strategies that include surgery, chemotherapy, and radiation therapy have proven effective across diverse malignancies. Variation in cancer cells is most impactful for molecularly targeted therapies, where these targetable molecules and mutations that are essential for tumor growth and progression may be entirely absent from some tumor types or patients, limiting the broad application of such therapies. For example, the BRAF V600E missense mutation is present in approximately 7% of cancer patients, but approximately 60% of melanoma^1^. Thus, the use of the selective BRAF V600E inhibitor Vemurafenib requires knowledge of the specific genotype of patient cancer cells for its application^2^. For this reason, understanding the scope of responsive tumors is critical to translation of each new therapy.

In theory, there can be much more consistency between the non-cancer cells that make up the tumor stroma and immune infiltrate, since these are normal cells lacking the unique mutational patterns of cancer cells. For this reason, the stroma has been described as the ‘Achilles heel’ of tumors, and stromal targeting encompasses established therapies such as Bevacizumab targeting VEGFA across a range of malignancies^3^, as well as immunotherapies. Immunotherapy represents a distinct type of non-cancer targeting, aiming to regulate the interaction between infiltrating immune cells and both the cancer cells and non-cancer cells in the growing tumor. In this way antibodies blocking molecules such as PD1 or CTLA4 can control tumors even though they are not expressed on cancer cells.

In addition, it is now clear that the success and failure of conventional interventions is linked to their interactions with the immune cells and tumor stroma. For example, post-surgical survival of colorectal cancer patients treated with conventional adjuvant therapy is linked to the immune infiltrate of the tumor^4,5^. For the response to radiation therapy, a range of innate sensors expressed in immune cells such as TLRs, RIG-I, and STING detect the endogenous adjuvants released following radiation of cancer cells and help determine whether CD8 T cell responses are optimally incorporated to eliminate the surviving cancer cells, or whether CD8 T cell responses are not incorporated permitting regrowth of the tumor^6–10^. Of these sensor mechanisms, cGas recognition of extranuclear DNA^11^ generates cGAMP which in turn activates the STING sensor pathway. Activation of the STING pathway generates type I IFN, which has locoregional impacts on antigen processing and presentation, and can help mature dendritic cells for effective migration and cross-presentation in the TdLN. Impacts on antigen presentation and dendritic cell maturation provide essential links to adaptive immunity, supporting CD8 T cell killing of surviving cancer cells in irradiated tumors^6,7,12^. While STING expression in cancer cells can impact other cells in the tumor via type I IFN, STING expression in non-cancer cells is essential to tumor control following radiation in a range of models^9^. Thus, for this innate sensing mechanism to function cGas-generated cGAMP must be transmitted from irradiated cancer cells to STING-expressing non-cancer cells of the tumor stroma.

Enpp1 was identified as a phosphodiesterase that results in cleavage of cGAMP and can therefore reduce the availability of cGAMP for STING activation^13^. Importantly, Enpp1 expression by cancer cells can limit activation of the STING pathway by degrading cGAMP, resulting in decreased tumor control^14,15^. Thus, Enpp1 represents a potentially important target to optimize innate immune activation in the tumor following radiation therapy^14,16^. However, in studying the role of Enpp1 in anti-tumor immune responses, we observed that Enpp1 expression is inconsistent between tumor types, suggesting that Enpp1 targeted inhibitors may have restricted application. If cancer cell expression is essential, it may be necessary to test each patient for cancer cell expression of Enpp1 to justify this intervention.

The aim of this study is to understand the importance of Enpp1 expression in non-cancer cells of the tumor and how this impacts tumor control by radiation. In this work we identify that while cancer cells may not express Enpp1, cells of the tumor stroma in particular tumor-associated macrophages express Enpp1. Using both Enpp1 knockout mice and a novel Enpp1 inhibitor, we demonstrate that Enpp1 expression in the non-cancer cells limits tumor control following radiation. This activity is dependent on STING and IFNAR1 expression in non-cancer cells, and tumor destruction by CD8 T cells. Enpp1 expression may represent a general mechanism of inflammatory suppression in cancer therapy thereby expanding the range of tumors that can be treated by Enpp1 inhibition to tumor types where cancer cell expression of Enpp1 is rare. In addition, this work demonstrates that radiation is a strong partner for novel Enpp1 inhibitors that may have limited impact as single agents, but strongly enhance CD8-mediated tumor control by radiation therapy.

## Results

**Fig. 1:**
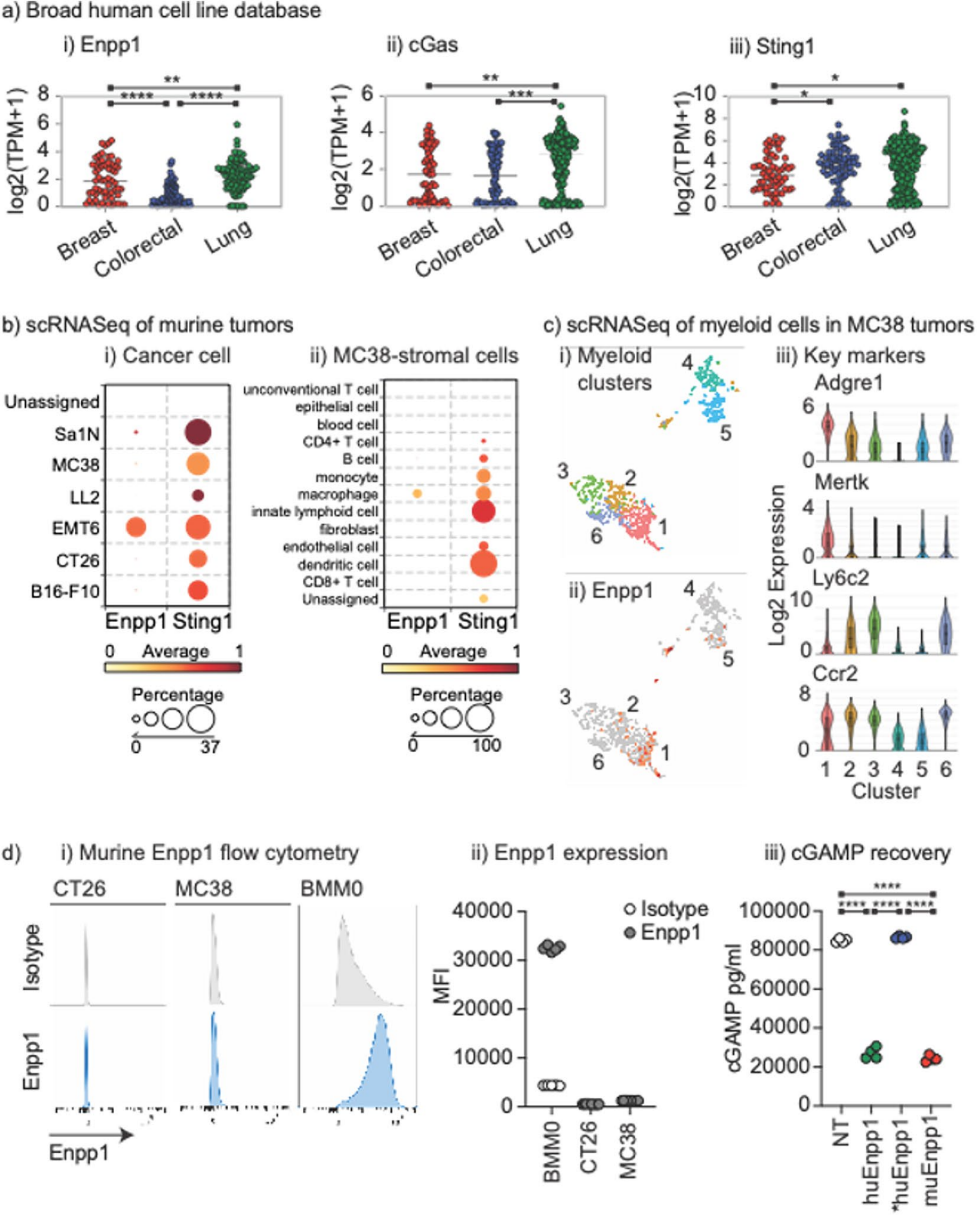
Colorectal cancer cells exhibit low or absent Enpp1 expression and require Enpp1 transfection to degrade cGAMP. (**a**) RNA expression of (i) Enpp1, (ii) cGas, and (iii) Sting1 in a panel of breast, colorectal, and lung adenocarcinoma cell lines present in DepMap portal. Each symbol represents one cell line, with expression as Log2 transcripts per million. (**b**) Analysis of scRNASeq of a panel of murine tumors highlighting Enpp1 and Sting1 expression in (i) Cancer cells and (ii) stromal cells in MC38 tumors. Scale shows degree of expression by color and the circle size shows the percentage of each cell type that expresses the gene. (**c**) Analysis of scRNASeq of CD45 + cells in MC38 tumors gated on myeloid cells. (i) TSNE plot showing 6 clustered myeloid populations from MC38 tumors. (ii) Enpp1 expression (red) in clustered populations. Iii) identification of clusters based on key gene expression. (**d**) (i) Flow cytometry for Enpp1 versus isotype control in murine CT26 and MC38 colorectal carcinoma cell lines, and bone marrow-derived macrophages (BMM0). (ii) Summary of MFI of (i) across replicates. (iii) Recovery of spiked cGAMP from MC38 cells (NT), MC38 cells stably transfected with human Enpp1 (huEnpp1), human Enpp1 T256A mutant (*huEnpp1), or murine Enpp1 (muEnpp1). Key: * *p* < 0.05; ** *p* < 0.01; *** *p* < 0.001; **** *p* < 0.0001.

We compared Enpp1 expression across a panel of cancer cell lines in the Broad Institute Cancer Dependency Map (DepMap) (https://depmap.org/portal/)^17^, and found that while breast cancer cell lines and lung cancer cell lines had a relatively high expression of Enpp1, colorectal cancer cell lines had Enpp1 expression that was significantly lower than either (Fig. 1a). The ability to generate cGAMP as measured by cGas expression was not significantly different between breast and colorectal cancer cell lines but was significantly lower than in lung carcinoma cell lines. Finally, the ability to respond to cGAMP as measured by Sting1 expression was similar between colorectal cancer and breast cancer, but both express less Sting1 than lung carcinoma cell lines. These data suggest that in colorectal cancer, while cGAMP production and sensing may be similar, Enpp1-mediated degradation of cGAMP in the tumor environment may be decreased. Notably, some cancer cells from each tumor origin lack cGas and/or Sting1 and so may be deficient in innate sensing following radiation, consistent with observations in other cancers^18^. Poor Sting1 expression in tumors has been associated with poor prognosis, though Sting1 expression in tumor stroma has shown a greater correlation with patient prognosis^19^. However, this is inconsistent across cancers with Sting1 and cGas expression showing no association with prognosis in most tumor types^20^. It may be that the baseline expression of STING pathway genes is less relevant if there is an absence of stimuli in tumors, and Enpp1 may play a role in this immune biology.

Enpp1 can also be expressed by immune cells in the tumor environment. Enpp1 expression has been described on germinal center B cells and plasma cells^21,22^, and T cells^21^. In macrophages Enpp1 expression may be linked to their differentiation^23^. To profile Enpp1 expression in cell populations infiltrating murine tumors, we analyzed a scRNASeq dataset of six murine tumor models^24^. This includes the mammary carcinoma cell line EMT6 and the colorectal carcinoma cell lines MC38 and CT26, and we analyzed cancer cells, stroma, and infiltrating immune cells^24^. Consistent with human colorectal carcinoma, Enpp1 was not significantly expressed in the CT26 or MC38 cancer cells in vivo (Fig. 1bi). Only EMT6 mammary carcinoma cancer cells expressed Enpp1 in agreement with human data that Enpp1 is more abundant in breast cancer cells. Notably, the lung cancer cell line LLC did not express Enpp1; however, analysis of lung cancer cell lines from human tumors demonstrated that these generally express Enpp1 (Fig. 1a). To determine whether LLC was an outlier, we analyzed Enpp1 gene expression in a published dataset of murine lung cancer cell lines^25,26^and confirmed that LLC did not express Enpp1 but 3 other lung cancer cell lines did express Enpp1 (Supplemental Fig. 1). To profile Enpp1 expression across a more diverse panel of murine cancer cell lines, we interrogated the TISMO resource^27^. This dataset shows that as with our prior analysis, our two colorectal cancer cell lines MC38 and CT26 did not express Enpp1 and LLC did not express Enpp1. The two breast cancer cell lines we had previously identified 4T1 and EMT6 expressed Enpp1, but a third E0771 did not (Supplemental Fig. 1). Additionally, while this dataset profiles B16 as expressing Enpp1 (Supplemental Fig. 1), the highly metastatic B16-F10 subclone is not shown to express Enpp1 (Fig. 1b). There may be some issues in this comparison between the results of two different research studies since in two different direct comparisons Enpp1 expression was not found to be significantly different between B16 and B16-F10 using microarray^28^ or RNASeq^29^. Nevertheless, these data demonstrate that there is variation between murine cell lines and as with human tumors, murine cancer cells can variably express Enpp1.

In contrast to the restricted expression of Enpp1, the ability to respond to cGAMP via Sting1 expression was widely shared between cancer cells and infiltrating cell types in this dataset (Fig. 1bii). Expression of Enpp1 was primarily observed in macrophages in MC38 tumors (Fig. 1bii). To explore the myeloid subtypes that express Enpp1 in tumors in more depth, we analyzed our previously published scRNASeq of CD45^+^ tumor infiltrating cells in MC38 tumors^30^. Myeloid sub-populations were selected by expression of Itgam and re-clustering generated 6 subpopulations of myeloid cells (Fig. 1**ci**). Notably, Enpp1 was predominantly expressed in cluster 1 cells (Fig. 1**cii**), and these cells were particularly characterized by expression of Adgre1 (F4/80), Mertk, and absence of Ly6c2 (Fig. 1ciii), suggesting that macrophages are the dominant myeloid population expressing Enpp1 in these tumors. Together these data suggest that macrophages in tumors express Enpp1 and this may limit cGAMP availability and subsequent immune activation even in tumors where cancer cells do not express Enpp1.

We confirmed the scRNASeq data by flow cytometry for Enpp1 in the murine colorectal cancer cell lines MC38 and CT26 (Fig. 1d). These cells poorly expressed Enpp1, in contrast bone marrow-derived macrophages strongly expressed Enpp1 (Fig. 1di-ii). In addition, MC38 cancer cells fail to degrade cGAMP spiked into cell culture (Fig. 1diii). Transfection of MC38 cells with a human or murine Enpp1 resulted in degradation of cGAMP, while transfection with the human Enpp1 T256A mutant^31^ did not (Fig. 1diii). Together, these data confirm a lack of Enpp1 protein and function in the cancer cells, supporting the RNASeq-based data. These data demonstrate that colorectal carcinoma cell lines lack Enpp1 expression and may exhibit decreased Enpp1 degradation of cGAMP in the tumor environment.

**Fig. 2:**
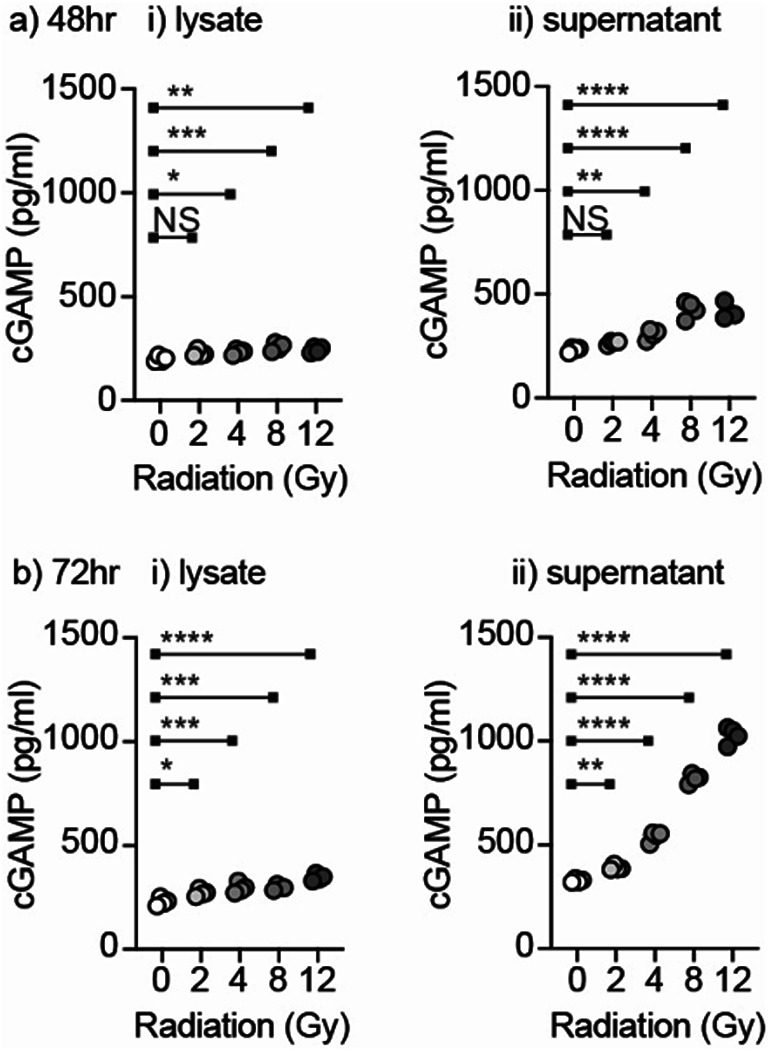
Radiation results in dose dependent release of cGAMP. MC38 cells were treated with a range of radiation doses and harvested after (**a**) 48 h or (**b**) 72 h to assess cGAMP in (i) the cell lysate or (ii) the cell supernatant. Key: NS = not significant * *p* < 0.05; ** *p* < 0.01; *** *p* < 0.001; **** *p* < 0.0001.

Cancer cells including CT26 and MC38 can endogenously generate cGAMP following radiation therapy, via cGAS sensing of radiation-induced micronuclei^11^. Following a range of RT doses delivered to MC38 cells in vitro, we observed a radiation dose-dependent production of cGAMP, which was present inside the cell lysates but was most detectable in the cell supernatant at 72 h (Fig. 2). These data confirm that RT treatment results in an endogenous source of cGAMP, which has the potential to impact neighboring cells. In the absence of cancer-cell associated Enpp1, degradation of cGAMP released from the cancer cells may be impacted by the presence of Enpp1-expressing macrophages in the tumor immune environment.

**Fig. 3:**
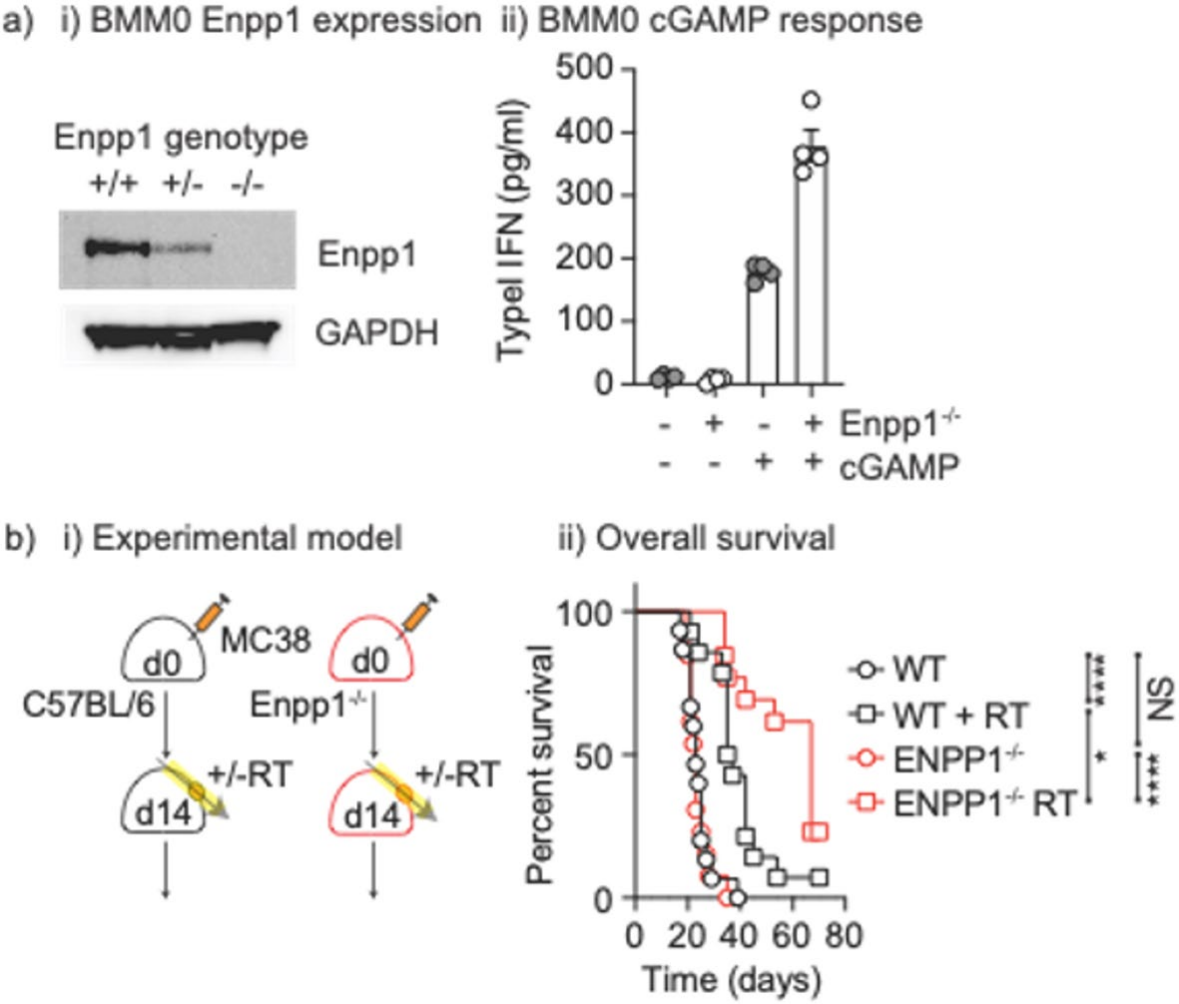
Enpp1 knockout results in increased responses to cGAMP and improved responses to radiation treatment. (**a**) (i) Bone marrow macrophages (BMM0) were generated from wt, Enpp1+/- or Enpp1-/- mice and cell lysates were western blotted for Enpp1 and GAPDH protein expression. Original blots are presented in Supplementary Figure 6. (ii) wt or Enpp1-/- BMM0 were left untreated or treated with cGAMP and type I IFN secretion was determined by bead assay. (**b**) (i) MC38 cells were injected into wt or Enpp1-/- mice and tumors were allowed to develop for 10-14 days. Mice were randomized to receive no further treatment or 12Gy focal RT to the tumor. (ii) Mice were followed for survival. Key: NS = not significant * p<0.05; ** p<0.01; *** p<0.001; **** p<0.0001.

To test the impact of Enpp1 expressed by non-cancer cells on the response to radiation therapy we analyzed Enpp1^−/−^ mice^32^. Loss of Enpp1 in these mice was confirmed by western blotting for Enpp1 in bone marrow macrophages derived from wild-type, heterozygous, or homozygous Enpp1^−/−^ mice (Fig. 3a.i.). To determine whether this impacted the sensitivity of these cells to cGAMP treatment, wt or Enpp1^−/−^ were treated with cGAMP and their production of type I IFN was measured. While wild-type macrophages produce type I IFN in response to cGAMP stimulation, Enpp1^−/−^ macrophages make a significantly stronger response (Fig. 3aii). To test the importance of non-cancer cell Enpp1 expression in the response to radiation therapy, MC38 tumors were implanted into wt C67BL/6 or Enpp1^−/−^ mice and, once established, mice were randomized to 12 Gy of radiation therapy delivered to the tumor by CT guidance or no further treatment (Fig. 3bi). In control, untreated mice tumor growth was not significantly different between wt and Enpp1^−/−^ mice. As anticipated, radiation therapy increased survival in wt mice (Fig. 3bii); however, radiation therapy was significantly more effective in Enpp1^−/−^ mice. These data demonstrate that host Enpp1 limits the response to radiation therapy. Importantly, the effect of Enpp1 loss was not observed without radiation therapy, demonstrating that radiation-induced cGAMP release is essential for host Enpp1 expression to be impactful. These data extend the range of tumors that can be impacted by Enpp1-targeted therapies to include those that lack Enpp1 in the cancer cells.

**Fig. 4:**
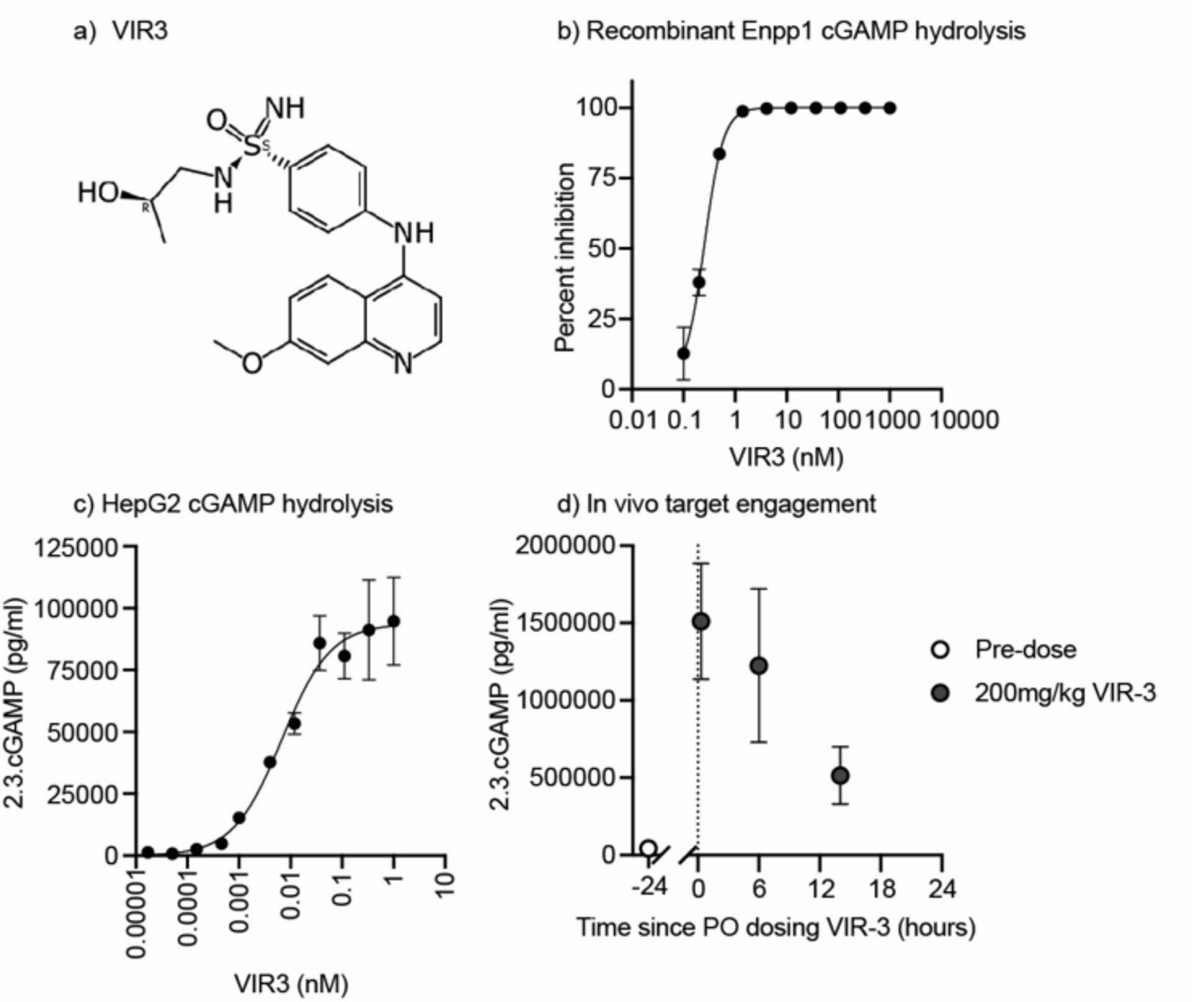
Development of VIR3 Enpp1 Inhibitor(**a**) Structure of VIR3. (**b**) Representative data from biochemical assay monitoring recombinant ENPP1 cGAMP hydrolysis activity and inhibition by VIR3. (**c**) Representative data from cellular assay monitoring cGAMP hydrolysis on HepG2 cells in the presence of VIR3. (**d**) In vivo target engagement was measured by administering 5ug of 2’3’-cGAMP IV to mice and then collecting blood samples 3 minutes later into a tube containing ENPP1 inhibitors. The level of cGAMP remaining the plasma at the time of collection was measured by ELISA both before VIR3 oral administration (open circles) or after (closed circles) (n = 3-5 mice per group, data is plotted as mean +/-SEM).

To test whether Enpp1-targeted therapies could improve the response to RT in wild type mice where the cancer cells do not express Enpp1, we developed a novel orally bioavailable Enpp1 inhibitor, VIR3. VIR3 is composed of a two-ring core structure with an aniline linker leading to a sulfonimidamide zinc chelator (Fig. 4a). The VIR3 target site is the N-pocket of ENPP1’s catalytic site, a similar location to the binding of other ENPP1 inhibitors^33^. VIR3 is a potent inhibitor of cGAMP hydrolysis in in vitro assays with an IC_50_ < 0.75 nM (Fig. 4b), which was the lower threshold for the biochemical inhibition assay using recombinant ENPP1. In cellular assays using HepG2 cells that express ENPP1 on their surface, VIR3 was able to protect spiked-in cGAMP from hydrolysis overnight with an EC_50_ of 6.7 nM (Fig. 4c). Comparing in vivo pharmacokinetics data from IV and PO routes of administration shows that VIR3 has a low oral bioavailability (Table 1). However, at high oral doses VIR3 is present in the plasma above the calculated mouse plasma adjusted EC_90_ for more than 8 h. To test the target engagement of VIR3 in vivo, we administered a small amount of cGAMP to mice either before or after oral gavage with VIR3. Plasma samples collected within 3 min of cGAMP administration showed that VIR3 was able to protect cGAMP from rapid hydrolysis in vivo (Fig. 4d). This effect was maximal closely following VIR3 administration and declined over time.

**Table 1:** In vivo PK of VIR3.

	Compound ID	VIR-3
I.V. 1 mg/kg	Cl (L/hr/kg)	1.66^#^
	Vss (L/kg)	52.5
	t1/2 (hr)	48.3
P.O. 60 mg/kg	AUC0-last, total (hr*ng/mL)	1420
	Cmax, total (ng/mL)	795
	t1/2 (hr)	24.3
	%F	5.7

^#^IV clearance is approximate given extrapolation from a portion of AUCinf>%20.

**Fig. 5:**
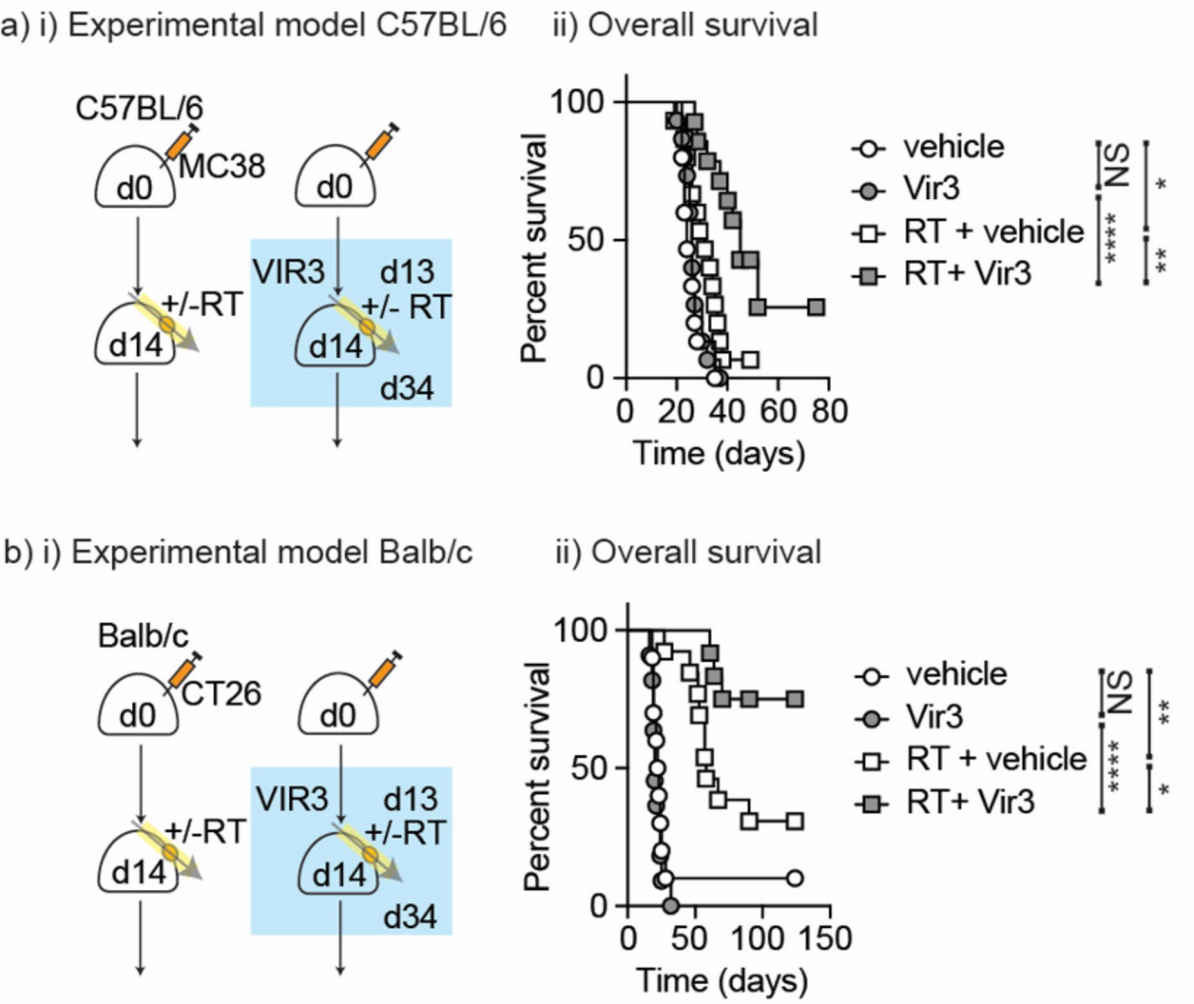
Treatment with VIR3 Enpp1 inhibitor improves tumor control by RT. (**a**) (i) MC38 cells were injected into wt mice and tumors were allowed to develop for 14 days. Mice were randomized to receive 21 daily doses of VIR3 or vehicle by oral gavage starting on d13, and further randomized to no further treatment or 12 Gy focal RT to the tumor on d14. (ii) Mice were followed for survival. (**b**) (i) Treatment as per a) but tumors were CT26 tumors injected into BALB/c mice (ii) Mice were followed for survival. Key: NS = not significant * *p* < 0.05; ** *p* < 0.01; *** *p* < 0.001; **** *p* < 0.0001.

To evaluate the novel Enpp1 inhibitor as a cancer therapy, C67BL/6 mice were implanted with MC38 tumors and once established, mice were randomized to receive daily oral gavage of the VIR3 Enpp1 inhibitor. Oral dosing was continued daily for 21 days, and mice were additionally randomized to receive 12 Gy radiation on the second day of dosing (Fig. 5ai). Mice were followed for tumor growth. While VIR3 did not impact tumor growth alone, it significantly improved the response to radiation therapy (Fig. 5aii). These data demonstrate that VIR3 inhibition of host Enpp1 improves tumor control following radiation therapy. In addition, the fact that radiation therapy is needed to see an impact in Enpp1^−/−^ mice or in mice treated with VIR3 suggests that the effect is dependent on radiation-induced cGAMP release. To confirm these data in an alternate murine model on a different genetic background, we used the CT26 colorectal carcinoma model which similarly lacks Enpp1 expression (Fig. 1) in BALB/c mice. Wild-type BALB/c mice were implanted with CT26 tumors and once established mice were randomized to receive daily oral gavage of the VIR3 Enpp1 inhibitor starting on day 13. Oral dosing was continued daily for 21 days, and mice were additionally randomized to receive 12 Gy radiation on the second day of dosing (day 14) (Fig. 5bi). As with MC38 tumors, while VIR3 did not impact tumor growth alone, it significantly improved the response to radiation therapy (Fig. 5bii). These data show that VIR3 improves the response to radiation therapy in two Enpp1-negative colorectal carcinoma models.

**Fig. 6:**
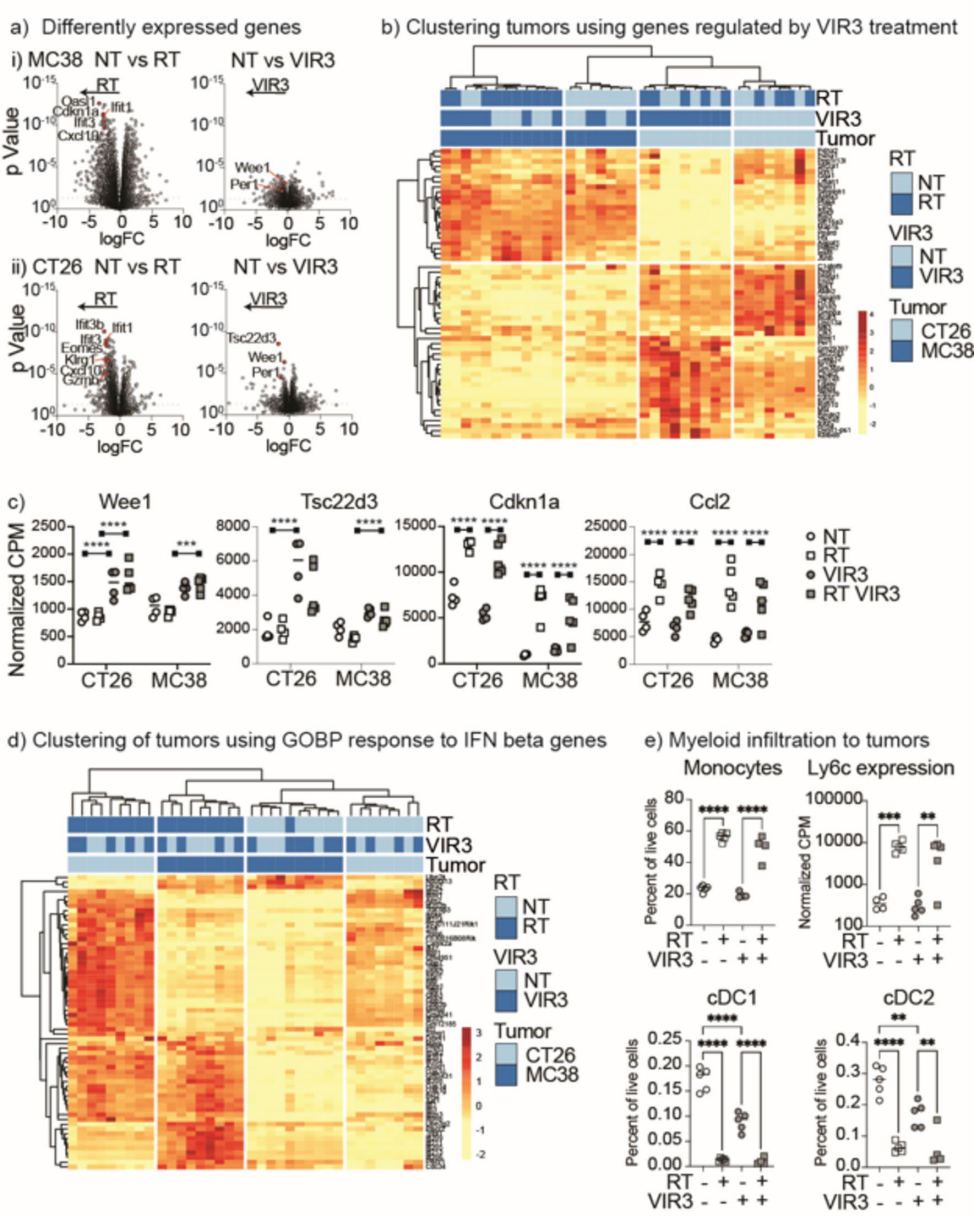
Impact of RT and VIR3 treatment on tumor gene expression. MC38 tumors were established in C57BL/6 mice and CT26 tumors were established in BALB/c mice. Mice were randomized to receive daily doses of VIR3 or vehicle by oral gavage starting on d13, and further randomized to no further treatment or 12 Gy focal RT to the tumor on d14. 4 days following radiation (d18) tumors were harvested and total gene expression in the tumors was analyzed by RNASeq, with 4 tumors for each treatment condition and tumor (32 total). (**a**) Volcano plots of differential gene expression shown as log fold change by pValue by treatment group. For (i) MC38 or (ii) CT26 tumors the graphs show differential gene expression due to RT alone, or VIR3 treatment alone. A selection of relevant genes are highlighted in red. (**b**) Expression of genes significantly regulated with FDR correction by VIR3 treatment in untreated (NT vs. VIR3) or irradiated tumors (RT vs. RT + VIR3) were used for cluster analysis. Blue colors indicate samples in each treatment group and the color scale yellow to red shows the degree of expression of each gene with unit variance scaling applied to each gene. (**c**) Expression of selected genes in each sample, by normalized CPM. Each symbol represents one tumor. (**d**) Expression of genes represented in the GOBP response to IFN beta genesets across samples. Blue colors indicate samples in each treatment group and the color scale yellow to red shows the degree of expression of each gene with unit variance scaling applied to each gene. (**e**) Flow cytometry of tumors treated as in a-d) to assess myeloid infiltration. Graphs show each cell type as a percent of all live cells in the tumor. Each symbol represents one tumor. Gene expression of Ly6c2 is also shown as a comparator, by normalized CPM. Each symbol represents one tumor. Key: * *p* < 0.05; ** *p* < 0.01; *** *p* < 0.001; **** *p* < 0.0001.

To understand the pathways activated in the tumor following radiation therapy and VIR3 inhibition of Enpp1, we performed RNASeq of the tumors 4 days following radiation treatment. We performed differential expression gene analysis between each treatment combination in each tumor. The volcano plots of differential gene expression highlight that a much greater number of genes were regulated by radiation therapy alone than by VIR3 treatment alone (Fig. 6a), and tumors treated with radiation + VIR3 regulated more genes than VIR3 alone (Fig. 6a). These data demonstrate that radiation has a larger effect on gene expression in the tumor than VIR3 treatment. To focus on the impact of VIR3, we identified genes that were significantly regulated by VIR3 treatment (NT vs. VIR3 or RT vs. RT + VIR3) with FDR correction (Supplemental Table 1). Unsupervised clustering of tumor samples using these genes was able to cluster VIR3-treated tumors (Fig. 6b), though there was a different pattern of response between CT26 and MC38 tumors. Wee1 was one of the few genes with expression significantly regulated with FDR correction by VIR3 treatment in both untreated and irradiated tumors in both CT26 and MC38 tumors (Fig. 6ci) (Wee1 MC38 NT vs. VIR3 pvalue 0.000857227 adjusted pvalue 0.295314135) CT26 NT vs. VIR3 pvalue 4.87E-07 adjusted pvalue 0.003488436 (MC38 RT vs. RT VIR3 pvalue 3.24E-09 adjusted pvalue 4.64E-05) CT26 RT vs. RT VIR3 pvalue 1.12E-07 adjusted pvalue 0.001607356. Wee1 is a G2 checkpoint kinase and prevents entry into mitosis following DNA damage in cancer cells^34,35^. Wee1 inhibition in combination with radiation can increase radiosensitivity by preventing DNA repair^36^. Similarly, Tsc33d3 is upregulated following VIR3 treatment in both tumors (Fig. 6cii). Tsc33d3 – also known as GILZ, is upregulated by glucocorticoids^37^ and downregulated by IFN exposure^38^, and can protect T cells against TCR-mediated apoptosis^37^. By contrast, genes such as Cdkn1a which encodes a well characterized cell cycle regulatory protein, and Ccl2 which encodes a myeloid-recruiting chemokine are not impacted by VIR3 treatment and as expected are induced by radiation (Fig. 6ciii-iv).

We applied Gene Set Enrichment Analysis to compare the impact of treatment across MC38 and CT26 tumors. There were a range of pathways that were significantly enriched by VIR3 treatment compared to untreated tumors, but little in common between the response to VIR3 treatment between the two different tumor types analyzed (Supplemental Fig. 2, Supplemental Table 2). Of interest, in MC38 tumors three of the gene sets with the largest effect size have a core overlapping set of genes including TCR constant genes Trbc1 and Trbc2, the TCR coreceptor genes CD4, CD3e, CD3d, CD3g, and TCR signaling genes CD247 and Lck (Supplemental Fig. 2c). While the gene set showed enrichment, these genes individually were not significantly regulated by VIR3 (Fig. 6). However, these data suggest potential immune regulation in the tumor by VIR3 treatment. To understand the additional impact of VIR3 inhibition on radiation, we compared gene sets enriched between tumors treated with RT versus RT + VIR3. There was a range of pathways that were significantly enriched by VIR3 treatment, but again there was little in common between the two tumor types analyzed (Supplemental Fig. 3). By contrast, radiation therapy alone upregulated a range of pathways in both MC38 and CT26 tumors, with the response to type I interferons highly enriched in both tumor types analyzed. Similarly, the combination of radiation and VIR3 treatment upregulated gene sets involved in the response to type I interferons compared to untreated tumors and compared to tumors treated with VIR3 alone in both tumor types analyzed (Supplemental Figs. 4–5). For this reason, a gene set associated with the response to IFN was able to delineate tumors treated with RT or RT + VIR3 from NT or VIR3 controls (Fig. 6d). In preclinical models, induction of type I IFN is necessary for tumor control by radiation therapy via impacts on hematopoietic cells in the tumor^39^. In turn this Type I IFN response is required for T cell expansion and recruitment to irradiated sites^40^. Given that Enpp1 can impact type I IFN produced by macrophages (Fig. 3), it may be that VIR3 inhibition of Enpp1 may alter the duration rather than the peak of the IFN response in tumors, explaining the improved outcome with VIR3 treatment. These data demonstrate that while there are some unique features associated with VIR3 treatment, at this timepoint radiation is the dominant impact on gene expression in the tumor and generates a proinflammatory response consistent with innate immune activation in the irradiated tumor environment^6,7^.

To validate the gene expression changes we used flow cytometry to determine cellular infiltration in MC38 tumors (Supplemental Fig. 6). At the same time point that we observed an RT-driven increase in Ccl2 expression in the tumor (Fig. 6c), we also observed a dramatic increase in CD11b^+^Ly6C^+^CD24^−^ monocytes infiltrating the tumor (Fig. 6ei), representing a wave of infiltration of immune cells with varied potential to differentiate in the tumor environment. This matches an increase in expression of the Ly6c2 gene observed by RNA analysis (Fig. 6eii). By contrast cDC1 (Ly6C^−^MHCII^+^F4/80^−^CD24^+^CD103^+^) and cDC2 (Ly6C^−^MHCII^+^F4/80^−^CD24^+^CD11b^+^) are decreased in the tumor following radiation (Fig. 6eiii-iv), consistent with the radiation-mediated maturation and migration we have previously observed in this tumor in response to the innate adjuvants released following radiation^30,41^. However, it is notable that VIR3 alone can cause DC loss, suggesting that single agent treatment may be helping to mature DC despite a limited single agent impact on tumor growth. These data show that radiation and VIR3 treatment result in changes in the cellular composition of the tumor consistent with their proposed mechanisms of action.

**Fig. 7:**
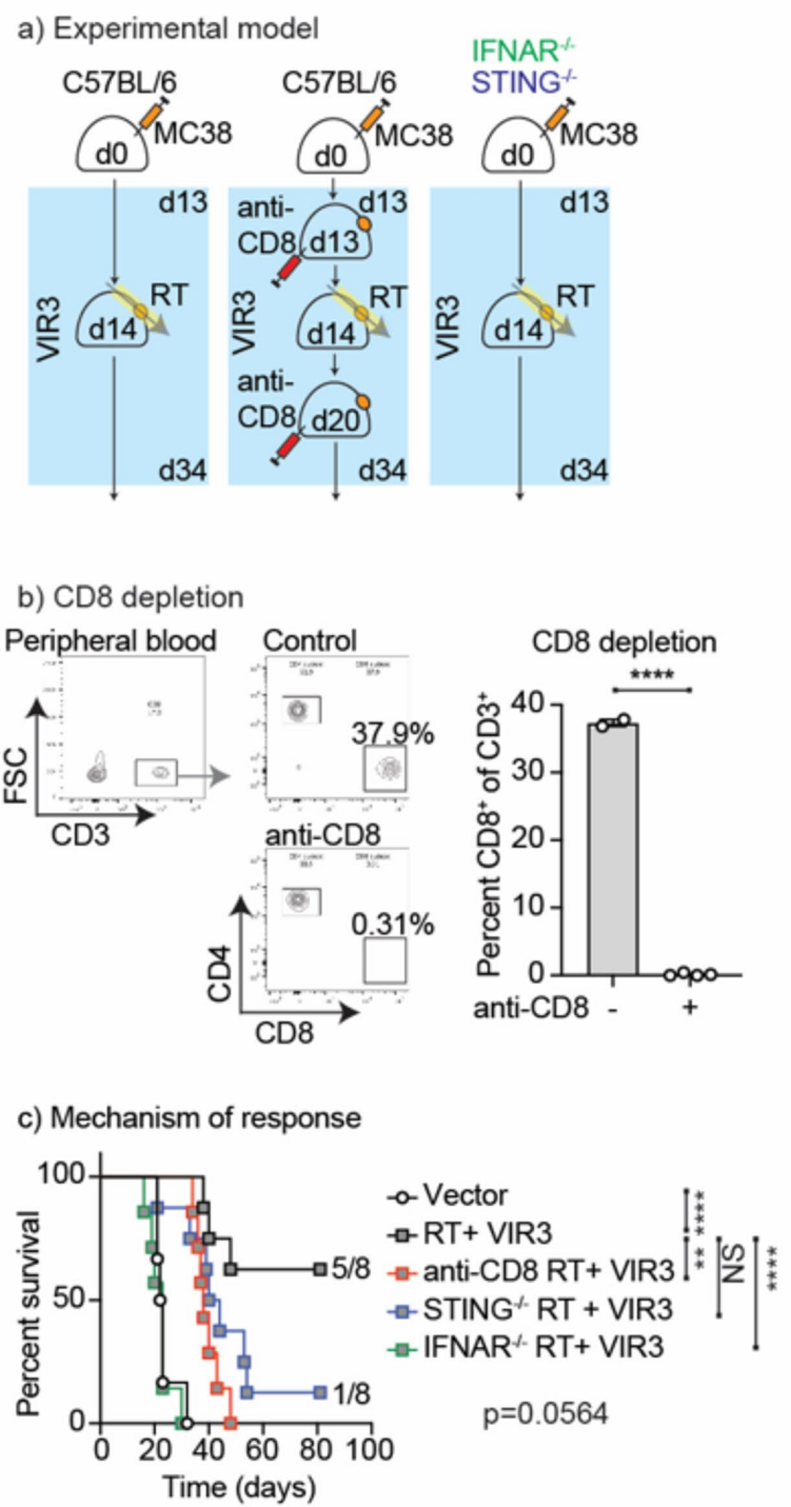
Mechanisms of tumor control by RT and VIR3. (**a**) MC38 tumors were established in wt, IFNAR1^−/−^, or STING^−/−^ mice. Mice were randomized to receive daily doses of VIR3 or vehicle by oral gavage starting on d13 and 12 Gy focal RT to the tumor on d14. A group of mice receiving combined RT + VIR3 were also treated with 3 weekly doses of anti-CD8 depleting antibodies starting on d13. (**b**) The impact of anti-CD8 on the depletion of CD8 T cells in the peripheral blood was assessed. Graphs show representative flow cytometry of peripheral blood gating on CD3 + cells and subgating for CD4 and CD8 T cells. The graph shows quantification of CD8 T cells between treatment and control groups. (**c**) Overall survival of mice treated as in (**a**). Key: NS = not significant * *p* < 0.05; ** *p* < 0.01; *** *p* < 0.001; **** *p* < 0.0001.

To validate the importance of innate sensing and IFN response mechanisms of tumor control by Enpp1 inhibition combined with radiation therapy, we compared radiation therapy and VIR3 treatment in MC38 tumors grown in wild-type C57BL/6 mice. STING^−/−^ mice, and IFNAR1^−/−^ mice (Fig. 7). As discussed above, the Type I IFN response is also required for T cell expansion and recruitment to irradiated sites^40^. For this reason, additional control cohorts of treated mice were given anti-CD8 the day prior to radiation and again 1 week later to deplete CD8 T cells during the response to treatment (Fig. 7). Importantly, we found that CD8 depletion abrogated tumor control following radiation therapy and VIR3 treatment, (Fig. 7) demonstrating that CD8 T cells remain the final effector mechanism in this system, consistent with our experience with exogenous STING ligands and radiation therapy^42^. Loss of STING in host cells also abrogated tumor control with fewer mice cured by RT plus VIR3, though there was not a statistically significant difference in survival by logrank test (Fig. 7). Loss of IFNAR1 in host cells completely abrogated all responses to treatment (Fig. 7). Together, these data demonstrate that treatment with RT and VIR3 to inhibit Enpp1 results in tumor control via an immune mechanism that is dependent on STING signaling in host cells and type I IFN responses in host cells.

## Discussion

Expression of Enpp1 is inconsistent between cancer cells. However, even where cancer cells lack Enpp1, stromal expression of Enpp1 and particularly macrophage Enpp1 expression can degrade cGAMP and result in decreased cGAMP availability for innate activation. In the present study we demonstrate that radiation causes cGAMP release, and that Enpp1 expression by non-cancer cells limits immune control of tumor by radiation. Using a novel Enpp1 inhibitor that can be delivered orally and results in on-target inhibition of Enpp1 activity in mice, we demonstrate that Enpp1-targeted therapy improves tumor control by radiation therapy in these cancers. While inhibition of Enpp1 regulates a range of gene pathways in tumors, radiation has a dominant impact on gene expression in the tumor immune environment, including increased activation of IFN-regulated gene pathways. Importantly, tumor control by radiation therapy combined with Enpp1 inhibition is dependent on STING and IFNAR1 expression in host cells, demonstrating that response functions via host sensing of cGAMP. This stromal Enpp1 may be critical in blocking cGAMP reaching the immune cell rich tumor stroma where there is high hematopoietic cell expression of STING. Cells of the tumors stroma require STING expression to contribute to the response to radiation therapy^9,43^, therefore cGAMP must pass from cancer cells to stromal cells and in doing so must traverse the macrophage-rich tumor-stromal boundary^44^. This has the potential to be applicable regardless of cancer cell expression of Enpp1, thus Enpp1 expression by the tumor stroma may limit tumor control following radiation across a broad range of patients and tumor types.

Exogenously administered STING ligands have shown significant activity as a cancer therapy in preclinical models^45–47^ and in combination with radiation^42^. Exogenously delivered STING plus radiation responses also rely on STING expression in non-cancer cells in the tumor^42^. However, responses to STING ligands generated endogenously via radiation therapy have a mixed dependency, with some studies showing that non-cancer cell expression of STING is essential^9^, and others that cancer cell STING expression is essential^8^. Similarly, in some studies cGas expression in DC is necessary for immune responses, while in other studies cGas expression in cancer cells is necessary^8,9^, meaning that the origin and therefore transmission of cGAMP is of unclear importance. It is possible that Enpp1 expression may play a role here, since those models discussed above that are dependent on cGAS and STING in the cancer cell are high Enpp1 expressing breast cancer cell lines^8,14^. Despite responses in murine studies, the clinical translation of exogenously administered STING ligands has been limited^48,49^. The pharmacodynamics of STING ligands, despite engineering to minimize phosphodiesterase cleavage, shows an extremely short in vivo half-life^49^. At present with short in vivo half-lives, STING ligands will only achieve pulsatile activity of the STING pathway following each administration. We observed that the dramatic tumor control following administration of STING ligands was mediated by a TNFa-mediated hemorrhagic necrosis^42^, consistent with prior data using DMXAA^47^. It is possible that different kinetics may prioritize type I IFN activity with a stronger transition to adaptive immune responses than TNFa-driven necrosis. It is reasonable to assume that endogenously generated cGAMP will similarly have a very short half-life in vivo, resulting in tight temporal regulation of STING pathway activation following radiation. Since Enpp1 degradation of cGAMP contributes to this short cGAMP half-life, it remains to be determined whether this regulatory pathway has a larger impact on STING signaling in humans when compared to mice. While the Enpp1 inhibitor VIR3 has shown high selectivity and specificity for Enpp1, additional studies are necessary to formally confirm target selectivity and off-target effects in vivo. Moreover, the clinical use of radiation as a multimodality therapy centered around fractionated chemoradiation combinations means that clinical translation may require a series of compromises compared to the preclinical design^50^. cGAMP production is radiation dose-dependent^11^, and the multiparameter impact of chemotherapy on Enpp1 activities and stromal immunity, and the impact of repeated re-irradiation on immune mechanisms^51^, remain to be determined in the use of Enpp1 inhibitors. At present the Enpp1 inhibitor has low oral bioavailability and high oral dosing results in drug present in the plasma above the EC_90_ for 8 h. This means that there is a substantial period of each day where Enpp1 may be partly or fully functional. Further chemical development is possible, but it would also be reasonable to incorporate VIR3 into extended-release formulations with the potential to sustain inhibition to ensure a more complete suppression of Enpp1. While toxicity was not observed, it may also be possible to formulate Enpp1 inhibition for locoregional release, as we have used for post-surgical delivery of STING ligands^52^.

Several different Enpp1 inhibitors have activity in pre-clinical models^15,53^ and clinical translation of some of these inhibitors is ongoing^53^. In our system Enpp1 inhibitors do not greatly impact tumor growth alone, but their activity is revealed following radiation therapy. This is consistent with a dependence on cGAMP production, which in the model investigated here is driven by radiation therapy. Alternative stimuli to produce cGAMP such as PARP inhibitors are plausible partners for Enpp1 inhibitors^54,55^. In addition, through a combined impact on cancer cell and stromal Enpp1, patients with low Enpp1 expression may be more responsive to PD1 inhibition as part of multimodality therapy^56^. In addition to its role in cGAMP degradation, the degraded nucleotides generated by Enpp1 can also be considered as a source of intermediates for further modification that can result in purinergic signaling in immune cells, which can be immune suppressive^53,55^. However, in relative quantities cleaved cGAMP may be significantly less abundant than metabolic sources of GMP and AMP. Given that the impact of Enpp1 blockade is lost in mice lacking STING expression, this shows that while relief of purinergic signaling could play a role, it is not sufficient to improve outcomes if the endogenous STING response is not present.

Based on our data we hypothesize that macrophages are the dominant cell type that express and secrete Enpp1 to limit locoregional STING activation in the tumor. Macrophages are consistent obstacles to immune activation in tumors^57–59^, in part via secretion of suppressive factors. Notably, macrophage secretion patterns are linked to their existing differentiation – thus macrophages pre-differentiated with IFNg or with IL-4 make distinct responses when stimulated with the same TLR4 ligands^60^. Thus, while macrophages are potent expressors of innate sensors, unless the immune environment in which they differentiate is known, the macrophage response to innate stimuli is not predictable. In addition, macrophages that are actively phagocytic can clear antigenic material released from dying cancer cells^61,62^. It has been broadly hypothesized that it would be preferable to allow antigenic material to reach the less abundant DC, which unlike macrophages can cross-present antigen to CD8 T cells. Moreover, it is critical that this antigen is accompanied by innate adjuvants that mature DC^9,41^. Using fluorescence tracking of dendritic cells in the tumor using the Kaede photoconvertible mice, we have shown that following radiation therapy exposure to endogenous innate adjuvants DC migrate to the TdLN via a CCR7-dependent mechanism^30,41^. This maturation and migration of DC is consistent with our observations of DC loss in tumors following radiation with or without Enpp1 inhibition. In the absence of CCR7-mediated DC migration radiation fails to generate a T cell-mediated extension of survival following radiation^30^. This mechanism of adjuvant-induced DC migration has the potential to help expand tumor-specific T cells in the TdLN, contributing to the CD8 T cell-dependent control of tumors treated with RT and VIR3 following the return of T cells to the irradiated tumor. Phagocytic macrophages expressing and secreting Enpp1 may limit the availability of both antigen and adjuvant to dendritic cells, which can result in a failure of CD8 T cell tumor control and contribute to the immunosuppressive phenotype of tumor environments^63^. As a fundamental part of the tumor stroma that is shared across many tumor types, tumor-associated macrophages form part of the “Achilles heel” of tumors. Blocking macrophage phagocytosis has resulted in improved tumor control over a range of targets and tumor models^57,64–69^, and our data suggests that Enpp1 inhibition may be a general strategy to improve tumor immunogenicity and radioimmunogenicity^70^. While it is reasonable to first target cancer types such as breast cancer and lung cancer where there is both cancer and stroma expression of Enpp1, our data suggests that stromal Enpp1 is sufficient to limit adaptive immune control of colorectal tumors where Enpp1 is not present in the cancer cells. Therefore, we propose that combining Enpp1 targeting with other therapies may have the potential to increase the innate response to radiation therapy across a broad array of tumors.

## Methods

### Analysis of public datasets

mRNA expression of Enpp1, cGAS, and STING1 in a panel of human cell lines was downloaded from the Broad Institute Cancer Dependency Map (DepMap: (ttps://depmap.org/portal/)^17^ using the 2024Q2 public dataset^71^. The relative expression of each gene in cell lines derived from breast, colorectal, and lung adenocarcinoma were compared. scRNASeq of Enpp1, cGAS, and STING1 in a panel of murine tumors was analyzed using BbrowserX and Vinci software (BioTuring Inc., San Diego, CA, USA: https://academic.bioturing.com) using data sets published by Kumar et al., 2018^24^. Gene expression from a panel of murine lung cancer cell lines grown in vitro^25,26^ (GSE204918 and GSE100412) were imported and converted from FPKM to TPM for inter-sample comparison. For additional analysis of published murine cell line gene expression, Enpp1 expression was analyzed using TISMO^27^. Gene expression in untreated control samples was used for this analysis. scRNASeq analysis of Enpp1 expression in myeloid cell subtypes in MC38 tumors was performed using published scRNASeq of CD45^+^ tumor infiltrating cells^30^, analyzed using Loupe Browser (10X Genomics). Venn diagrams of shared genes were constructed using InteractiveVenn (https://www.interactivenn.net/)^72^.

### Animals and cell lines

Animal protocols were approved by the Earle A. Chiles Research Institute (EACRI) Institutional Animal Care and Use Committee (Animal Welfare Assurance No. D16-00526). Experiments were performed according to ARRIVE guidelines. Survival experiments were performed with 8–14 mice per experimental group, and mechanistic experiments with 4–6 mice per group. Blinding was not used during experimental procedures in part due to the evident impact of radiation therapy on treatment groups and distinct dietary requirements of Enpp1^−/−^ mice. For tissue harvest the mice were euthanized by CO2 inhalation followed by cervical dislocation. Experiments utilized 6–8-week-old C57BL/6 (#000664), IFNAR1^−/−^ (#028288), and Sting1^−/−^ (*Goldenticket* Stock# 017537) mice that were obtained from The Jackson Laboratories. Enpp1^−/−^ mice (#012810) were obtained from The Jackson Laboratories and maintained on an elevated magnesium diet to limit ectopic mineralization^73^. The MC38 colorectal carcinoma line^74^ was obtained from Dr. Kristina Young at EACRI. HepG2 cells were from ATCC. Cells were cultured in EMEM + 10% FBS, respectively. For cellular assays, media was exchanged with Charcoal-stripped FBS (Gibco cat.# 12676029) which does not exhibit intrinsic cGAMP hydrolysis activity. The CT26 murine colorectal carcinoma line^75^ was obtained from ATCC (Manassas, VA). Pathogen and mycoplasma contamination testing were performed on all cell lines using the IMPACT II Mouse PCR Profiling from IDEXX BioAnalytics.

To generate cell lines expressing Enpp1, the DNA nucleotides of open reading frame for human ENPP-1 wild type protein and T256A mutant were cloned into pTwist Lenti SFFV vectors by Twist Bioscience. Lentivirus were then generated by transfecting Lenti-X 293T cells (Takara) with these two constructs using TransIT Lentivirus System (Mirus). MC38 cells were transduced with *un-titered supernatant containing* Lentivirus to generate MC38-human ENPP-1 expressing cells, *with the single amino acid mutation in Enpp1 as the internal control*. The pUNO1 plasmid carrying the mouse ENPP1 gene was purchased from InvivoGEN. Following transfection positive clones were identified through selection using 200 µg/ml of blasticidin. The pool of MC38 transfectants was subjected to multiple sorting using FACSAria Fusion cell sorter (BD Biosciences) to select for high expressing clones. The expression of ENPP-1 on the surface of the transfected cells were confirmed by staining with fluorescent-labeled antibodies specific to ENPP-1 and flow cytometry analysis.

### cGAMP release and recovery

To quantify cGAMP production and release following radiation, a total of 5 × 10^4^ cells were plated in a 24-well plate and allowed to rest overnight before irradiation. Charcoal filtered FBS was used to create complete media cDMEM without phenol red for the growth of MC38 cells. All other cell lines were cultured in cRPMI supplemented with charcoal-filtered FBS and without phenol red. An CIX2 cabinet x-ray irradiator (Xstrahl) was used for ex vivo radiation of cells in culture. Supernatants were collected to assay cGAMP release. To harvest cell lysates, the culture media was replaced with 100 µL of M-PER (cat# 78501) obtained from ThermoScientific, following the manufacturer’s instructions. A 2’3’-cGAMP elisa kit (cat# K067-H1W/H5W, Arbor Assays) was used to measure cGAMP levels, following the manufacturer’s instructions. To quantify cGAMP degradation by cells in culture, cells were spiked with 100ng/ml of cGAMP (cat# tlrl-nacga23s, InvivoGen) and cultured overnight. cGAMP remaining in the supernatant was quantified using a 2’3’-cGAMP assay kit as above.

### VIR3 Enpp1 inhibitor characterization

Enzymatic inhibition assays were performed by incubating 1.5 nM ENPP1 (R&D Systems cat# 6136-EN-010) with serial dilutions of Vir-3 for 15 minutes at 37^o^C in buffer (50mM Tris pH 8.0, 250mM NaCl, 0.5mM CaCl_2_, 1uM ZnCl_2_, 1% DMSO) before adding 20 uM 2’3’-cGAMP (Invivogen cat.# tlrl-nacga23). The reaction was incubated for 30 min at 37^o^C prior to assessing the generated AMP using the AMP-Glo assay (Promega cat.# V5012). Cellular inhibition of cGAMP hydrolysis was performed using HepG2 cells pre-incubated with serial dilutions of Vir-3 in DMEM + 10% Charcoal stripped FBS for 30 min at 37^o^C with 5% CO_2_. 100 ng/mL of 2’3’-cGAMP was then added to the cells and incubated for 20 h at 37^o^C with 5% CO_2_. Media was then removed and the remaining cGAMP was quantified using a 2’3’-cGAMP ELISA kit (Cayman Chemical cat.# 501700).

For pharmacokinetic analysis, female C57BL6 mice were dosed with Vir-3 by either IV administration or oral gavage or IV administration either once or daily for 5 days. 3 mice were used per time point with staggered groups to cover all times. Blood was collected at 0.25, 0.5, 1, 3-, 5-, 8- and 24-hours post-dose into heparinized tubes. Plasma was prepared from each sample and protein precipitated prior to LC-MS/MS injection. Levels of Vir-3 were quantitated, and data was analyzed using Certara’s Phoenix Winnonlin.

To test pharmacodynamics / target engagement, 3–5 female 6–8-week-old C57BL6 mice were used for each time point tested. Animals were dosed P.O. with Vir-3 at time 0. 3 minutes prior to each sample collection mice were administered 5ug 2’3’-cGAMP by I.V. and then blood was collected at times indicated in the text directly into a tube containing a saturating amount of ENPP1 inhibitor. Plasma was isolated from each sample and then tested for the level of cGAMP present using a 2’3’-cGAMP ELISA kit (Cayman Chemical).

### Tumor treatments

Tumors were implanted subcutaneously into the right flank as follows: C57BL/6 2 × 10^5^ MC38; BALB/c 2 × 10^5^ CT26. When tumors were approximately 5 mm in average diameter at approximately 10–14 days following implantation, mice were randomized to receive treatment with CT-guided radiation using the Small Animal Radiation Research Platform (SARRP) from XStrahl. Dosimetry was performed using Murislice software from XStrahl. The SARRP delivered a single dose of 12 Gy to an isocenter within the tumor using a 10 mm x 10 mm collimator and a 45° beam angle to minimize dose delivery to normal tissues. The Enpp1 inhibitor VIR3 was delivered through oral gavage starting 1 day prior to RT daily for 21 days. Tumors were measured using calipers in two directions and reported as mean tumor diameter. Survival curves were based on an endpoint of tumor diameter of greater than 12 mm in any direction.

To deplete CD8 T cells, 200 µg of depleting anti-CD8b (53 − 5.8, BioXCell, West Lebanon, NH) were administered intraperitoneally. This administration took place one day before the first dose of Vir3, followed by two additional anti-CD8b administrations at 7-day intervals.

### Tissue processing and RNASeq analysis

Mice were implanted with tumors and randomized to treatment with 12 Gy RT and VIR3 as above. 4d following RT, the tumors were harvested, debulked and immediately flash frozen by rapid immersion in liquid nitrogen. Frozen tumors were crushed, treated with 500 µl of RNAlater, then frozen at -80^o^C. To extract RNA, the samples were thawed on ice, and 500 µl of buffer RLT (QIAGEN Cat. No. 79219) was added and samples were homogenized. Following centrifugation, RNA was isolated using a QIAGEN RNeasy Plus Mini Kit (Cat No. 74134), according to the manufacturer’s instructions. Quality was checked on nanodrop (ND-1000) and the quantity of RNA was determined on Qubit 4 flourometer. To prepare for sequencing, samples were processed using an Illumnia TruSeq^®^ Stranded mRNA Library Prep kit with Illumina TruSeq RNA Single Indexes Set A and Set B barcoding kits. An Illumnia NovaSeq 6000 S1 Reagent Kit v1.5 was used to make RNA libraries along with a NovaSeq XP 2-Lane Kit v1.5. RNA libraries were sequenced on NovaSeq 6000.

Demultiplexed fastq files for all samples were first processed with FastQC for general quality control. All the samples in the sequencing run passed read level QC with at least 17 million reads per sample. Average read depth across all samples were 51 million reads. Raw illumina BCL data was demultiplexed using Illumina bcl2fastq2 v2.20. Gene expression counts were quantified using salmon-v.1.1.0 ^76^ for all samples sequenced. Differential gene expression analysis was performed using the R software package edgeR^77^. Differential gene expression analysis was performed on all groups of Tumor+/-Drug+/-RT combinations. Gene set enrichment analysis was performed using GSEA v4.3.2 Preranked method^78,79^. The following genesets; c3.all.v2023.2.Hs.symbols.gmt, c5.all.v2023.2.Hs.symbols.gmt, c7.all.v2023.2.Hs.symbols.gmt from Molecular Signatures Database (gsea-msigdb.org) were used for enrichment analysis.

### Flow cytometry

For analysis of Enpp1 expression, bone marrow macrophages were prepared as previously described^59^. Briefly, bone marrow was cultured with 40 ng/mL recombinant murine CSF-1 (PeproTech), and media was replaced every 3–4 days. Macrophages or cancer cell lines were resuspended in PBS containing 2 mM EDTA and 2% BSA for 30 min with 100 µL of anti-ENPP1 PE (YEI/19.1 Biolegend), or anti-IgG2bk PE isotype control (ebMG2b, eBioscience). Flow cytometry was performed on 3–5 cell samples or cell suspensions from 3 to 5 independent murine tumors, and fluoresence minus one controls were used to identify gating thresholds. Data was acquired on a BD Fortessa flow cytometer and analyzed using FlowJo software from Tree Star, v10.8.

To quantify CD8 T cells in the peripheral blood of mice, blood was collecting in lithium heparin coated tubes (BD Microtainer cat. # 365965, BDBiosciences) and red blood cells were lysed with FACS lysing solution (cat. # 349202, BDBiosciences). Antibodies used for FACS analysis included anti-CD3 APC (17A2, eBiosciences) and anti-CD8a (53 − 6.7, eBiosciences) and anti-CD4 FITC (RM4-5, Invitrogen).

Analysis of tumor infiltrating myeloid cells was performed as previously described^30^. Briefly, following dissection, tumors were weighed and minced into small fragments, then transferred into C tubes from Miltenyi Biotec containing enzyme digest mix with 250U/mL collagenase IV (Worthington Biochemical, #LS004188), 30U/mL DNase I (Millipore-Sigma, #4536282001), 5mM CaCl_2_, 5% heat inactivated FBS and HBSS. Tissue was dissociated using a GentleMACS tissue dissociator from Miltenyi Biotech. This was followed by incubation at 37 °C for 30 min with agitation. Enzymatic reactions were quenched using ice cold RPMI containing 10% FBS and 2mM EDTA. Single cell suspensions were then filtered through 100 μm (tumor) or 40 μm (dLN) nylon cell strainers to remove macroscopic debris. Cells were washed and counted for staining. 2 × 106 cells were stained with Zombie Aqua Viability Dye from BioLegend (#423102) in PBS for 10 min on ice, then Fc receptors were blocked with anti-CD16/CD32 antibodies from BD Biosciences (2.4G2) for an additional 10 min. After centrifugation, the supernatant was removed and cell were stained with a surface antibody cocktail containing in FACS buffer (PBS, 2mM EDTA, 2% FBS) and Brilliant Stain Buffer Plus from BD Biosciences (#566385) for 20 min on ice. The following antibodies were purchased from BioLegend; F4/80-PerCP/Cy5.5 (BM8), CD11c-PE/Cy7 (N418), CD90.2-A700 (30-H12), CD19-A700 (6D5), MHC-II-BV421 (M5/114.14.2), CD11b-BV605 (M1/70), CD8a-BV650 (53 − 6.7), and Ly-6 C-BV711 (HK1.4). CD103-APC (2E9) and CD24-APC e780 (M1/69) were obtained from Thermo Fisher Scientific. CD45-BV786 (30-F11) was purchased from BD Biosciences. After surface staining, cells were washed in FACS buffer and fixed for 20 min on ice with Fixation/Permeabilization Buffer from BD Biosciences (#554722). All samples were resuspended in FACS buffer and acquired on a BD Fortessa flow cytometer. Data were analyzed using FlowJo software from Tree Star, v10.7. Monocytes in the tumor were gated as leukocytes/ single cells/ Live/ CD45+ /CD90.2-CD19- /Ly-6 C+ /CD24-. cDC1 in the tumor were gated as leukocytes/ single cells/ Live/ CD45+ /CD90.2-CD19- /Ly-6 C- /MHC-II+ /CD24 + F4-80- /CD11b- /CD103+. cDC2 in the tumor were gated as leukocytes/ single cells/ Live/ CD45+ /CD90.2-CD19- /Ly-6 C- /MHC-II+ /CD24 + F4-80- /CD103- /CD11b+.

### Western blotting

Bone marrow macrophages were obtained as described above and plated on 24 wells plates. After overnight incubation, cells were washed twice with cold PBS and lysed in Pierce RIPA Buffer (Cat#8990, ThermoFisher, Waltham, MA) supplemented with Halt Protease and Phosphatase Inhibitor Cocktail (Cat#78440, ThermoFisher, Waltham, MA). Protein concentrations were quantified using Pierce BCA Protein Assay Kit (Cat#23225, ThermoFisher, Waltham, MA). Samples were denatured at 95 °C in XT Sample Buffer 4×(Cat#1610791, Bio-Rad, Hercules, CA) and loaded onto 4–12% Criterion XT Bis-Tris Protein Gels (Cat#345 − 0124, Bio-Rad, Hercules, CA). Proteins were transferred onto PVDF Transfer Membrane (Cat#88518, ThermoFisher, Waltham, MA) and probed for Enpp1 (Cat#2061, Cell Signaling Technology, Danvers, MA), and GAPDH (Cat#2118S, Cell Signaling, Danvers, MA). Membranes were incubated with HRP-conjugated goat anti-rabbit IgG (Cat#31460, Invitrogen, Carlsbad, CA) as a secondary antibody and afterwards incubated with SuperSignal West Pico PLUS Chemiluminescent Substrate (Cat#34580, ThermoFisher, Waltham, MA). Chemiluminescence was detected with a ChemiDoc MP imager (Bio-Rad, Hercules, CA).

### Statistics

Data were analyzed and graphed using Prism from GraphPad Software (v9.0). Individual data sets were compared using Student’s T-test and analysis across multiple groups was performed using one-way ANOVA with individual groups assessed using Tukey’s comparison. Kaplan Meier survival curves were compared using a log-rank test.

## Electronic supplementary material

Below is the link to the electronic supplementary material.

Supplementary Material 1

## Data Availability

RNASeq data were deposited to the NCBI GEO under accession number GSE264328. All other data is present in the manuscript and supplemental figures.
