# Supplementary material for: A novel small molecule Enpp1 inhibitor improves tumor control following radiation therapy by targeting stromal Enpp1 expression
Source: Sci Rep. 2024 Dec 2;14:29913. doi: 10.1038/s41598-024-80677-8 (PMC11612208; doi:10.1038/s41598-024-80677-8)
Supplement: Supplementary file 1 — Supplementary Material 1 [file 41598_2024_80677_MOESM1_ESM.pdf]

Supplemental Figure 1

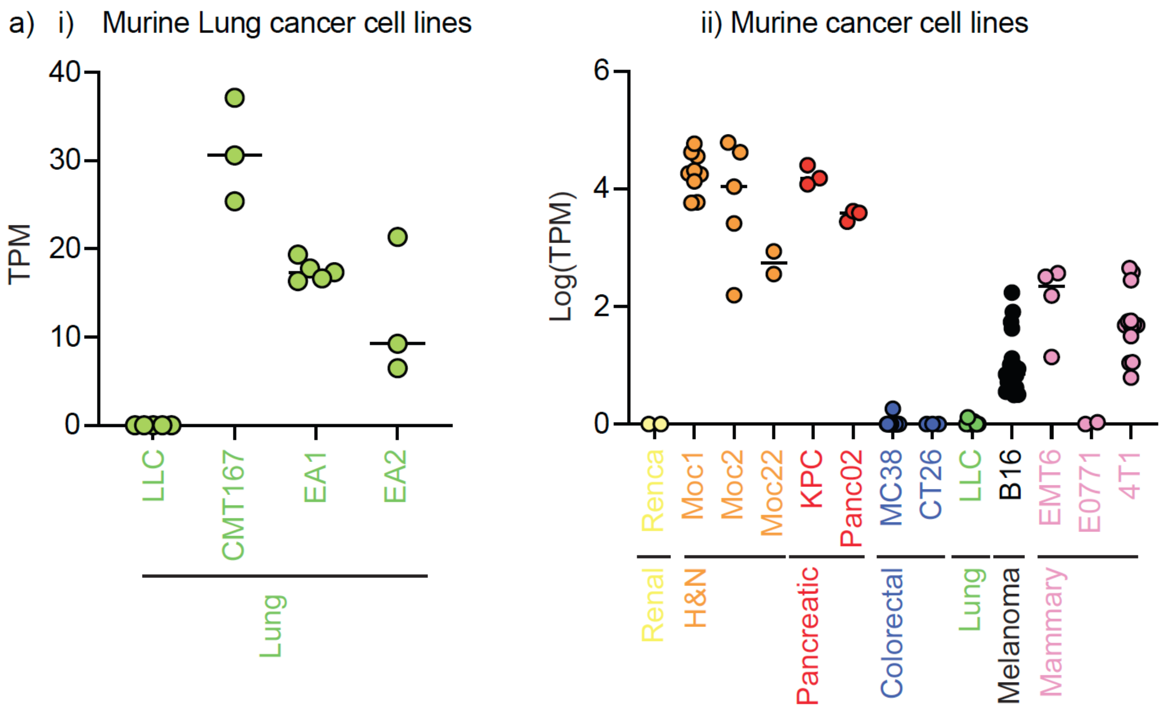

Supplemental Figure 1. a) i) Expression of Enpp1 in murine lung cancer cell lines from GSE204918 and GSE100412. ii) Expression of Enpp1 in control untreated murine cancer cell lines analyzed using TISMO. Cell lines are grouped and colored by cancer origin.

Supplemental Figure 2

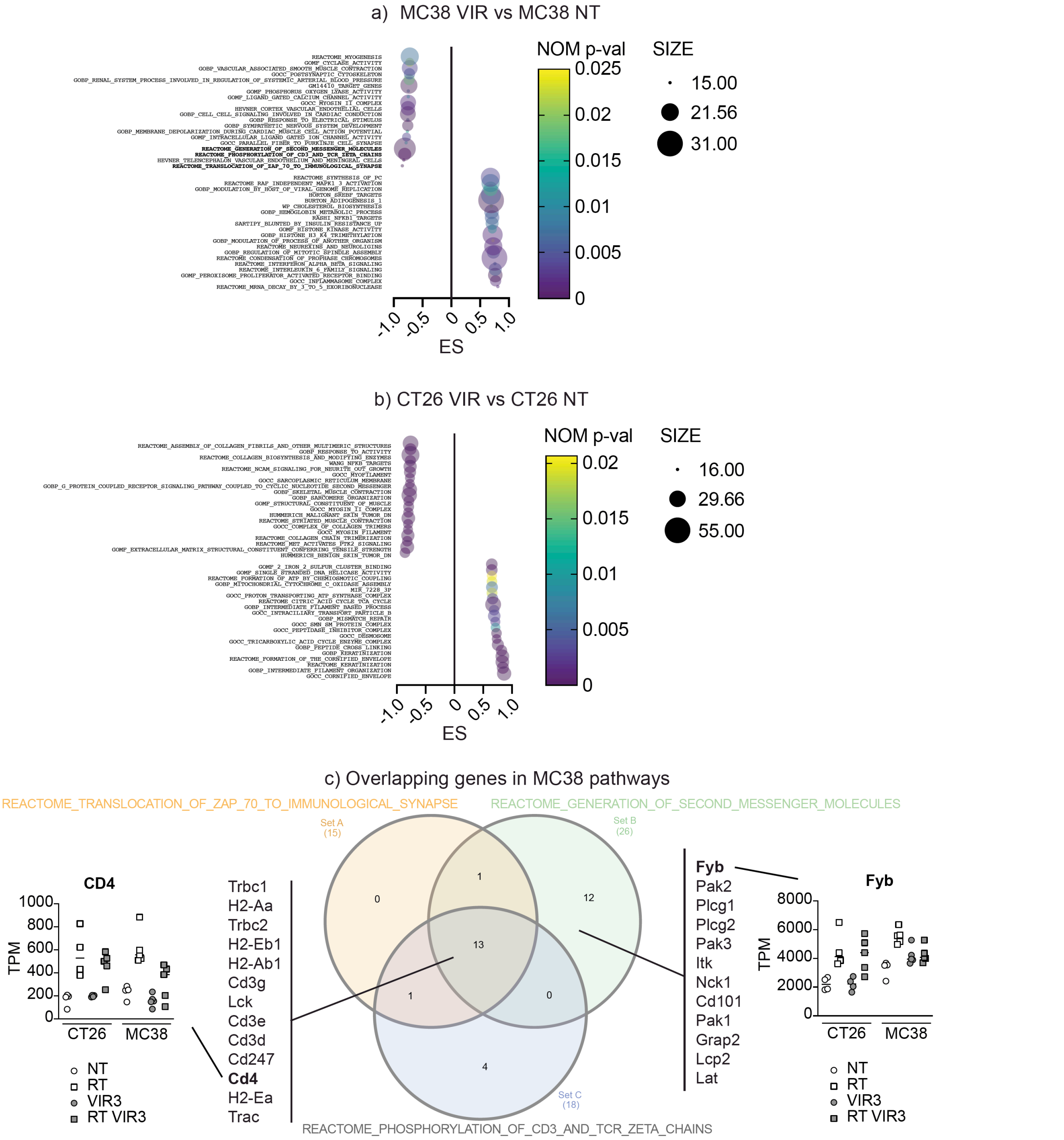

Supplemental Figure 2. GSEA results for NT vs VIR3 treatment in MC38 and CT26 tumors. Top 20 positive and negatively regulated pathways shown. Graph plots effect size on x-axis, nominal p-value in color scale, and the number of genes in the pathway is represented by the size of the circles. Potential immune-related pathways are highlighted in bold.

Supplemental Figure 3

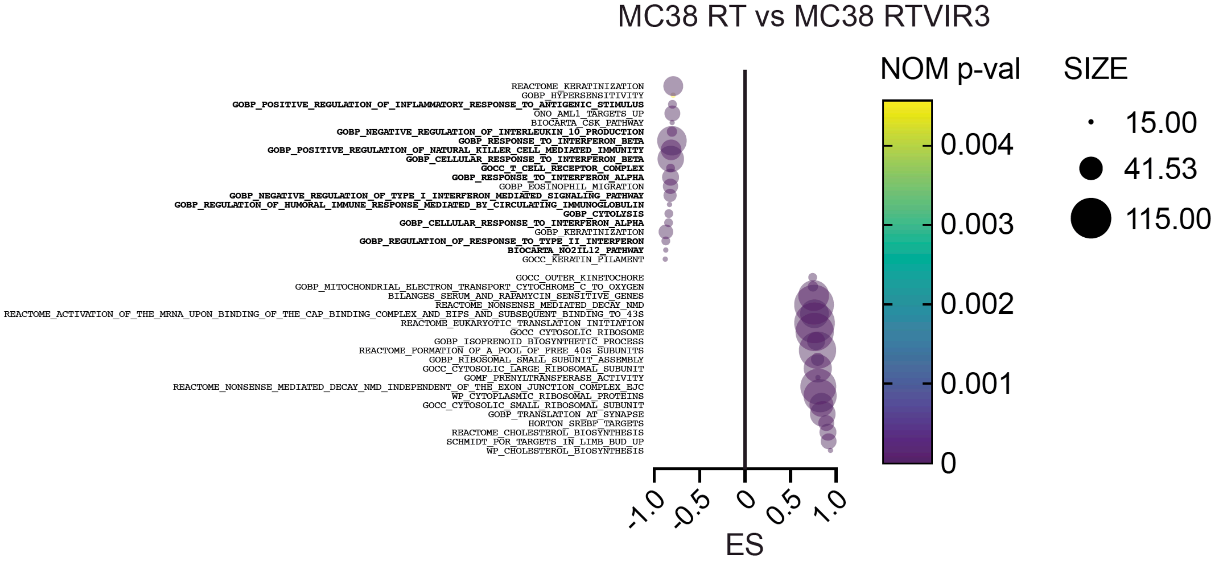

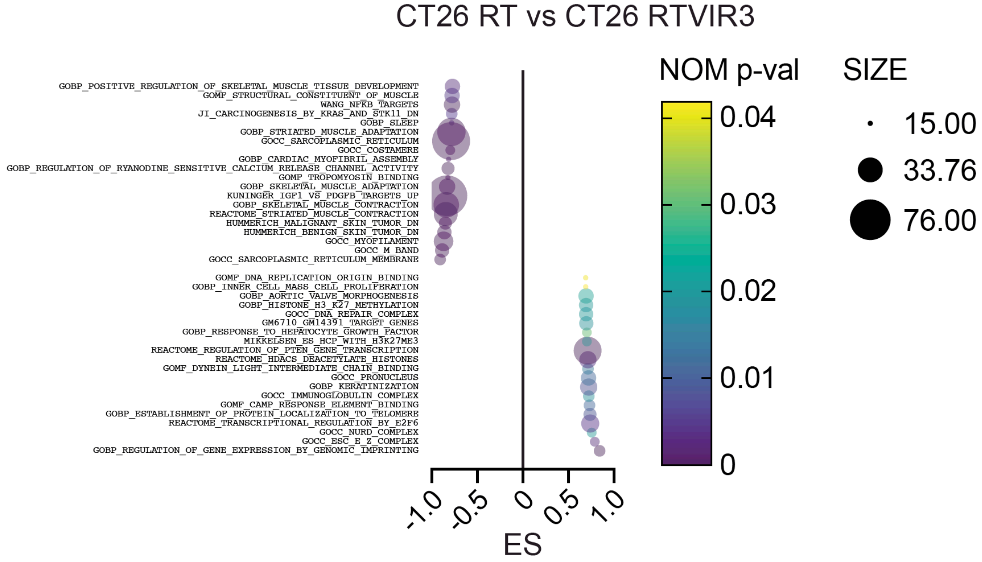

Supplemental Figure 3. GSEA results for RT vs RT+VIR3 treatment in MC38 and CT26 tumors. Top 20 positive and negatively regulated pathways shown. Graph plots effect size on x-axis, nominal p-value in color scale, and the number of genes in the pathway is represented by the size of the circles. Potential immune-related pathways are highlighted in bold.

Supplemental Figure 4

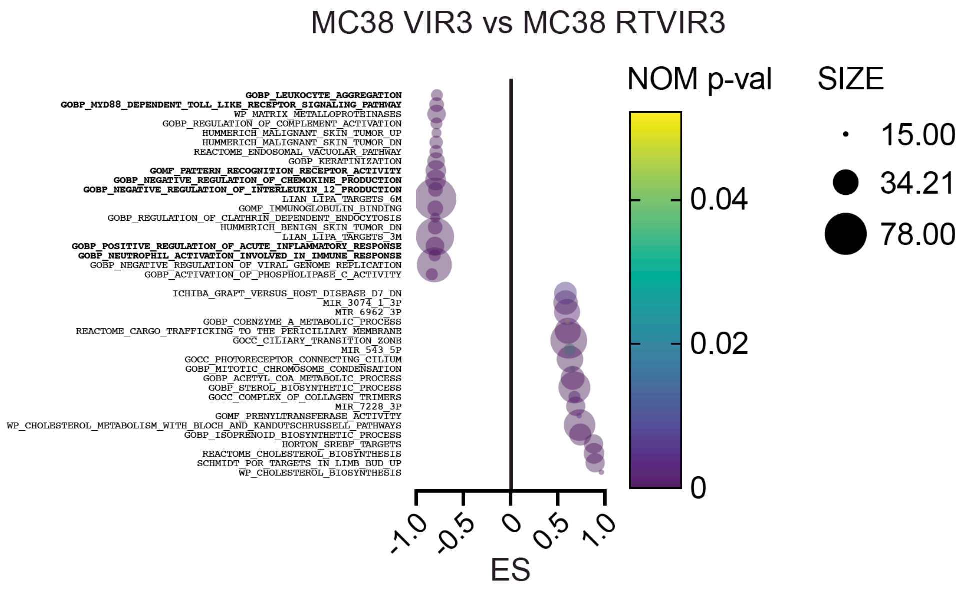

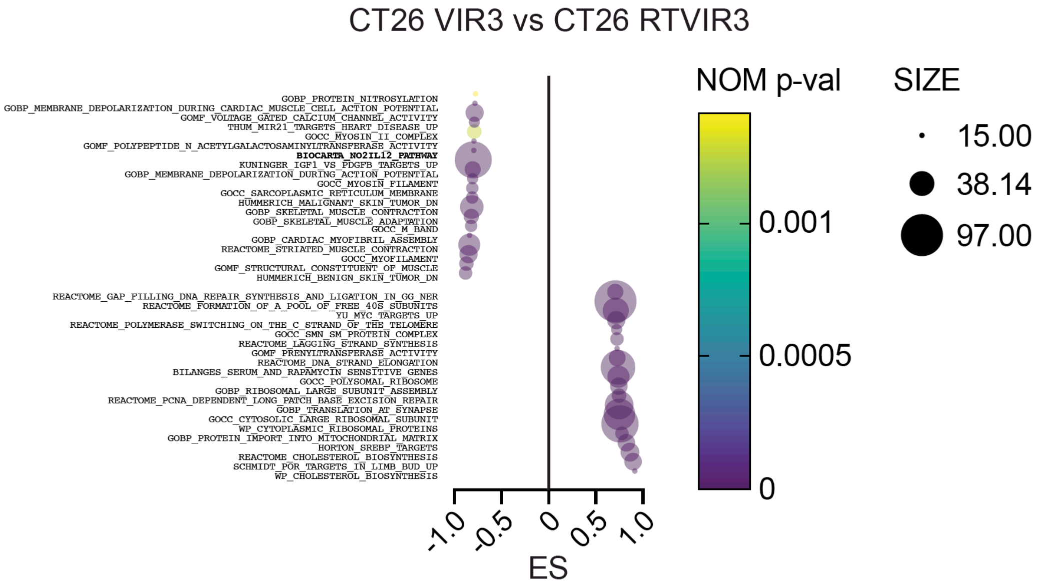

Supplemental Figure 4. GSEA results for VIR3 vs RT+VIR3 treatment in MC38 and CT26 tumors. Top 20 positive and negatively regulated pathways shown. Graph plots effect size on x-axis, nominal p-value in color scale, and the number of genes in the pathway is represented by the size of the circles. Potential immune-related pathways are highlighted in bold.

Supplemental Figure 5

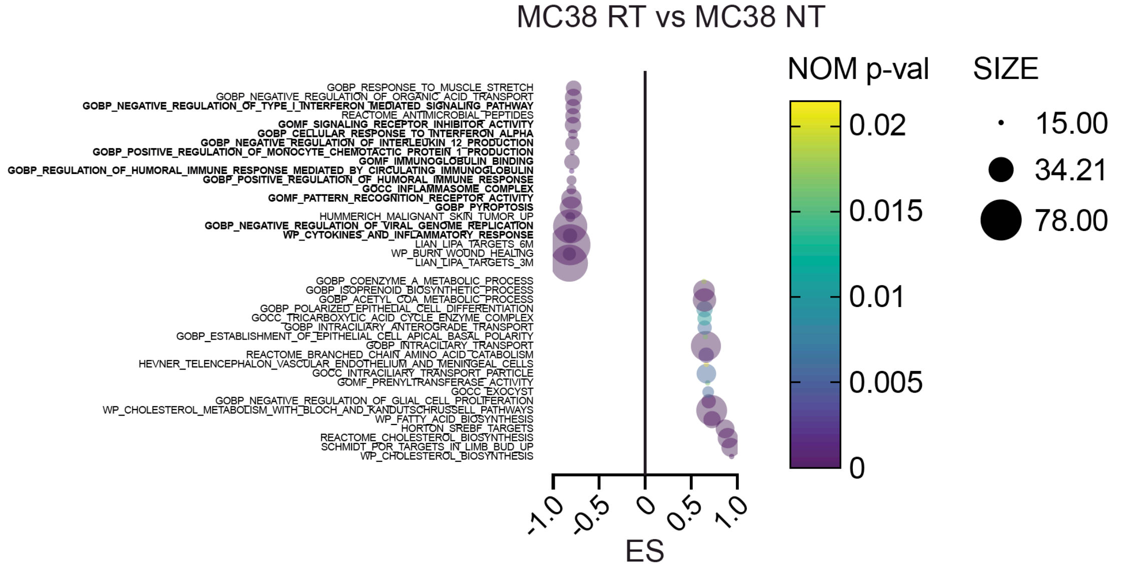

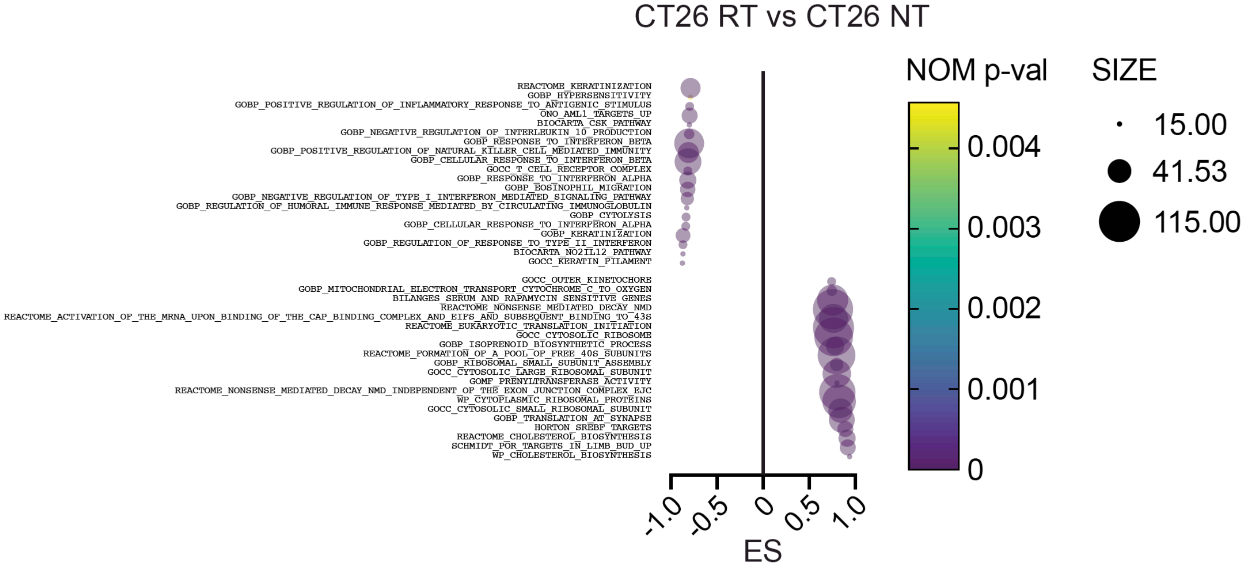

Supplemental Figure 5. GSEA results for NT vs RT treatment in MC38 and CT26 tumors. Top 20 positive and negatively regulated pathways shown. Graph plots effect size on x-axis, nominal p-value in color scale, and the number of genes in the pathway is represented by the size of the circles. Potential immune-related pathways are highlighted in bold.

Supplemental Figure 6

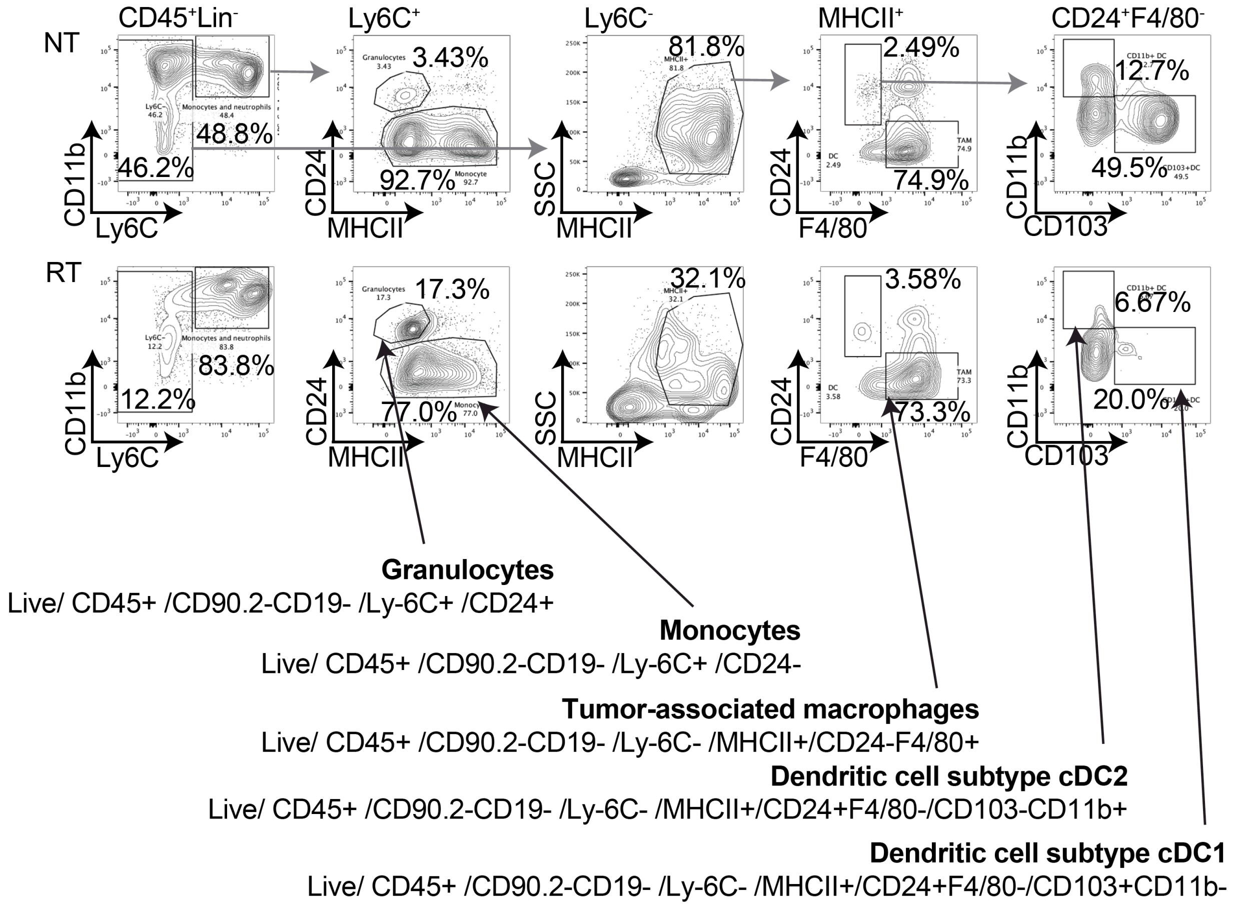

Supplemental Figure 6. Representative flow cytometry for myeloid cells infiltrating MC38 tumors that are untreated (NT – top) or irradiated at 12Gy (RT – bottom). Arrows (gray) show subgating to identify myeloid subtypes and (black) final populations for analysis.

Supplemental FIgure 7

Enpp1 +/+ +/- -/-

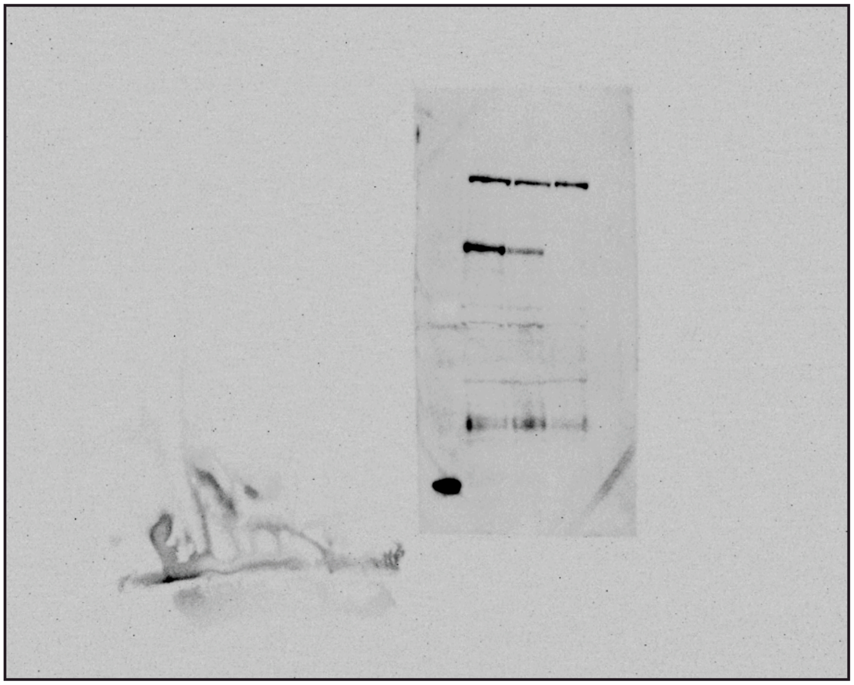

GAPDH +/+ +/- -/-

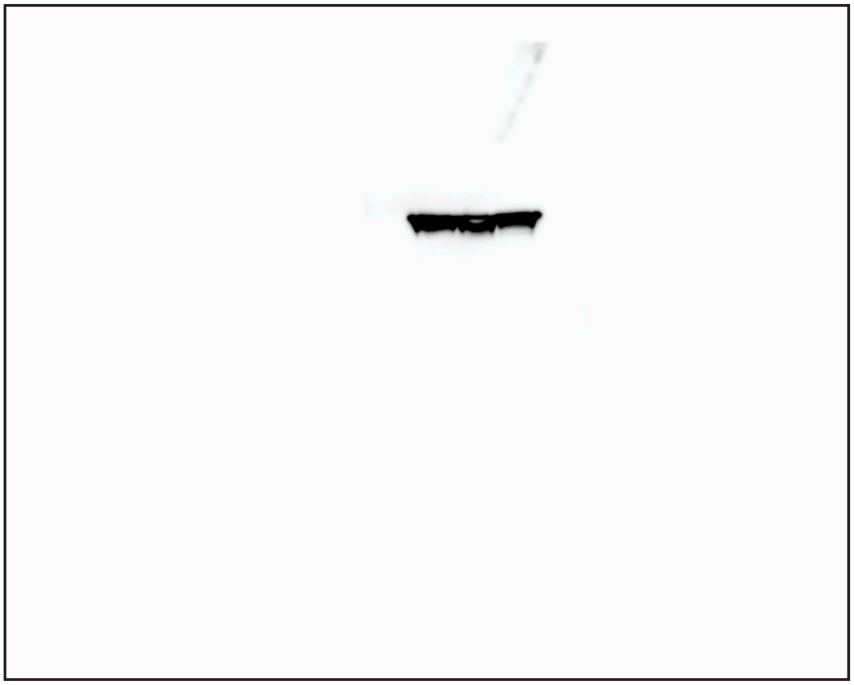

Supplemental Figure 7. Original uncropped western blots associated with Figure 3a.
